# Soluble high-molecular-weight amyloid-β species derived from amyloid-β-laden brains induce cerebral β-amyloidosis

**DOI:** 10.1093/braincomms/fcag188

**Published:** 2026-07-20

**Authors:** Mayu Kashiwagi-Hakozaki, Hirokazu Uchigami, Yasushi Naka, Asuka Kokawa, Tatsuo Mano, Junki Cho, Kaoru Yamada, Akinori Miyashita, Norikazu Hara, Takeshi Ikeuchi, Alberto Serrano-Pozo, Matthew P Frosch, Bradley T Hyman, Tatsushi Toda, Masashi Fukayama, Tetsuo Ushiku, Tomoko Wakabayashi, Takeshi Iwatsubo, Tadafumi Hashimoto

**Affiliations:** Department of Neuropathology, Graduate School of Medicine, The University of Tokyo, Tokyo 113-0033, Japan; Department of Pathology, Graduate School of Medicine, The University of Tokyo, Tokyo 113-0033, Japan; Department of Neuropathology, Graduate School of Medicine, The University of Tokyo, Tokyo 113-0033, Japan; Department of Neurology, Graduate School of Medicine, The University of Tokyo, Tokyo 113-0033, Japan; Department of Degenerative Neurological Diseases, National Center of Neurology and Psychiatry, Tokyo 187-8502, Japan; Department of Neuropathology, Graduate School of Medicine, The University of Tokyo, Tokyo 113-0033, Japan; Department of Neuropathology, Graduate School of Medicine, The University of Tokyo, Tokyo 113-0033, Japan; Department of Neurology, Graduate School of Medicine, The University of Tokyo, Tokyo 113-0033, Japan; Department of Degenerative Neurological Diseases, National Center of Neurology and Psychiatry, Tokyo 187-8502, Japan; Department of Neuropathology, Graduate School of Medicine, The University of Tokyo, Tokyo 113-0033, Japan; Department of Degenerative Neurological Diseases, National Center of Neurology and Psychiatry, Tokyo 187-8502, Japan; Department of Neuropathology, Graduate School of Medicine, The University of Tokyo, Tokyo 113-0033, Japan; Department of Molecular Genetics, Brain Research Institute, Niigata University, Niigata 951-8585, Japan; Department of Molecular Genetics, Brain Research Institute, Niigata University, Niigata 951-8585, Japan; Department of Molecular Genetics, Brain Research Institute, Niigata University, Niigata 951-8585, Japan; Department of Neurology, Alzheimer’s Disease Research Unit, Massachusetts General Hospital, Charlestown, MA 02129, USA; Department of Neurology, Alzheimer’s Disease Research Unit, Massachusetts General Hospital, Charlestown, MA 02129, USA; Department of Neurology, Alzheimer’s Disease Research Unit, Massachusetts General Hospital, Charlestown, MA 02129, USA; Department of Neurology, Graduate School of Medicine, The University of Tokyo, Tokyo 113-0033, Japan; Department of Pathology, Graduate School of Medicine, The University of Tokyo, Tokyo 113-0033, Japan; Department of Pathology, Graduate School of Medicine, The University of Tokyo, Tokyo 113-0033, Japan; Department of Neuropathology, Graduate School of Medicine, The University of Tokyo, Tokyo 113-0033, Japan; Department of Innovative Dementia Prevention, Graduate School of Medicine, The University of Tokyo, Tokyo 113-0033, Japan; Department of Neuropathology, Graduate School of Medicine, The University of Tokyo, Tokyo 113-0033, Japan; Department of Degenerative Neurological Diseases, National Center of Neurology and Psychiatry, Tokyo 187-8502, Japan; Department of Innovative Dementia Prevention, Graduate School of Medicine, The University of Tokyo, Tokyo 113-0033, Japan; Department of Neuropathology, Graduate School of Medicine, The University of Tokyo, Tokyo 113-0033, Japan; Department of Degenerative Neurological Diseases, National Center of Neurology and Psychiatry, Tokyo 187-8502, Japan; Department of Innovative Dementia Prevention, Graduate School of Medicine, The University of Tokyo, Tokyo 113-0033, Japan

**Keywords:** senile plaques, β-amyloidosis, propagation, oligomers, *in vivo* seeding

## Abstract

Spatiotemporal spreading of amyloid-β peptide deposition as senile plaques is a key pathogenic process in the brains of patients with Alzheimer’s disease; however, the molecular properties of amyloid-β strains that initiate the spreading of amyloid-β peptide as aggregation seeds *in vivo* remain poorly understood. In this study, we discovered that the intrahippocampal injection of soluble amyloid-β species with a molecular weight of >150 kDa isolated from the brains of plaque-laden amyloid-β precursor protein transgenic mice or patients with Alzheimer’s disease using size-exclusion chromatography, dramatically accelerated β-amyloidosis in the transgenic mice brains. In contrast, intrahippocampal injection of soluble amyloid-β species with 50–70 kDa or 10–20 kDa never induced β-amyloidosis. Moreover, injection of the soluble amyloid-β species with >150 kDa into cerebrospinal fluid of young transgenic mice via the cisterna magna predominantly induced amyloid-β deposition within the wall of leptomeningeal arteries surrounding the brain, reminiscent of cerebral amyloid angiopathy. The seeding activity of the soluble high-molecular-weight amyloid-β was prevented by the immunodepletion of amyloid-β and abolished by formic acid denaturation, suggesting that these amyloid-β oligomers are crucial in inducing β-amyloidosis. Furthermore, we have shown that the soluble high-molecular-weight amyloid-β is present in the brains of patients with Alzheimer’s disease and induced β-amyloidosis. These results indicate that the soluble high-molecular-weight amyloid-β oligomers may play an important role in the spatiotemporal spreading of amyloid-β deposition in Alzheimer’s disease brains.

## Teaser

Kashiwagi-Hakozaki *et al*. report that inoculation of soluble amyloid-β species with a molecular weight of >150 kDa induces cerebral β-amyloidosis. This seeding activity was abolished by formic acid denaturation. Soluble high-molecular-weight amyloid-β oligomers may therefore play an important role in the spatiotemporal spreading of amyloid-β deposition in Alzheimer’s disease.

## Graphical Abstract

**Graphical Abstract:**
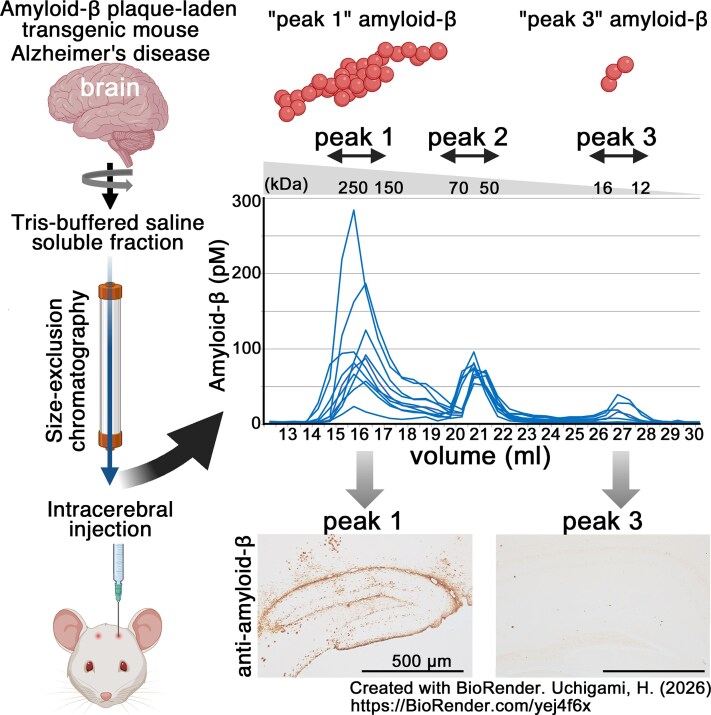

## Introduction

Massive deposition of amyloid-β peptide (Aβ) as senile plaques is a histopathological hallmark in the brains of patients with Alzheimer’s disease. Production of Aβ is mediated by β- and γ-secretase-dependent proteolytic processing of Aβ precursor protein (APP) in neurons. Based on several genetic and pathological studies, the widely accepted amyloid hypothesis postulates that Aβ accumulation in the brain is the trigger of a cascade of adverse events leading to Alzheimer’s disease.^1-3^ Deposition of Aβ is known to follow a spatiotemporal pattern with disease progression as described by the Thal amyloid phases.^4,5-7^ However, the molecular mechanisms that determine the pattern of Aβ deposition and its spreading in the brain are not fully understood. Although rodent Aβ does not exhibit spontaneous deposition in the brain, transgenic (tg) animal models expressing human APP with familial Alzheimer’s disease–linked mutations exhibit cerebral Aβ deposition in the brain.^8-11^ Several studies report that intracerebral inoculation of brain extracts from brains of patients with Alzheimer’s disease or plaque-laden APP tg mice induces β-amyloidosis in APP tg mice.^12-16^ Furthermore, it has been reported that Aβ as well as the prion protein accumulate in brains of patients with iatrogenic Creutzfeldt–Jakob disease.^17-19^ In contrast, Aβ protofibrils or fibrils pre-formed from synthetic Aβ polypeptides *in vitro* induce no or minimal Aβ deposition even at concentrations that are 1000 times higher than those found in the brain lysate,^13,20^ indicating that *in vitro* aggregated Aβ preparations do not contain the Aβ conformers needed to efficiently induce cerebral β-amyloidosis *in vivo*. These findings strongly suggest that Aβ seeds are present in Alzheimer’s disease brains or plaque-laden APP tg mouse brains and play important roles in Aβ deposition and its spatiotemporal spreading. However, the identity of these Aβ seeds responsible for Aβ deposition and spreading in the brain remains unclear.

In this study, we describe that the Tris-buffered saline (TBS)-soluble Aβ from brains of plaque-laden APP tg mice elutes into three fractions by the size-exclusion chromatography, at >150 kDa (Peak 1), 50–70 kDa (Peak 2) and 10–20 kDa (Peak 3) and that Aβ oligomers were present only in the Peak 1 fraction. Intrahippocampal injection of Peak 1 fraction induced a unique laminar pattern of Aβ deposition in the hippocampus of APP tg mice, whereas Peak 2 and Peak 3 fractions did not. Aβ deposition induced by Peak 1 fraction was abrogated by the immunodepletion of Aβ with anti-Aβ antibodies confirming that Aβ seeds present in Peak 1 fraction drive Aβ deposition. Denaturation of oligomers by pre-treatment with formic acid abrogated the ability to induce Aβ deposition, indicating that Aβ seeds are Aβ oligomeric species. Moreover, Peak 1 Aβ extracted from the brains of patients with Alzheimer’s disease induced a similar pattern of Aβ deposition in the brains of APP tg mice. These results indicate that the soluble high-molecular-weight Aβ species are the molecular culprit underlying the spatiotemporal spreading of β-amyloidosis in the Alzheimer’s disease brain.

## Materials and methods

### Human autopsied brains

Frozen blocks and formalin-fixed paraffin sections of autopsied brains from six patients with Alzheimer’s disease and five controls without dementia were provided by Drs. Matthew P. Frosch and Bradley T. Hyman from the Massachusetts Alzheimer’s Disease Research Center (MADRC) (NIH grant P30AG062421). All donors or their next-of-kin provided written informed consent for the brain autopsy and the study was conducted under the MADRC Neuropathology Brain Bank Institutional Review Board. All patients with Alzheimer’s Disease had pathologically confirmed Alzheimer’s Disease with Braak stages V or VI and none of the controls met neuropathological diagnostic criteria for any neurological disease. This study was approved by the Ethics Committee of the Graduate School of Medicine at the University of Tokyo (No. 11959).

### Animals

We used A7 tg mice overexpressing human APP with *Swedish* and *Austrian* familial Alzheimer’s Disease (FAD)-linked mutations (KM670/671NL + T714I) in neurons.^11^ C57BL/6J wild-type mice were purchased from Japan SLC, Inc. All animals were maintained on food and water *ad libitum* with a 12-h light/dark cycle. Mice of both sexes were used in this study, with a male-to-female ratio of 3:4 (60 males and 80 females; total number of animals, *n* = 140). All experiments were approved by the Institutional Animal Care and Use Committee of the Graduate School of Medicine at the University of Tokyo.

### Antibodies, immunoblotting and ELISA

Antibodies used included: monoclonal antibody 82E1 against human Aβ amino-terminal-end (IBL), monoclonal antibody BAN50 against human APP and Aβ (FUJIFILM Wako), monoclonal antibody 6E10 against human APP and Aβ (BioLegend), monoclonal antibody 4G8 against APP and Aβ (BioLegend), monoclonal antibody BA27 against Aβx-40 (FUJIFILM Wako), monoclonal antibody BC05 against Aβx-42 (FUJIFILM Wako), polyclonal antibody C43 against Aβx-43 (IBL), polyclonal antibody against pyroglutamated Aβ (N3pE, IBL), monoclonal antibody GA5 against Gfap (Merck), polyclonal antibody against Iba-1 (FUJIFILM Wako) and the polyclonal antibody against carboxy-most-terminal 13 amino acids of human TDP-43.^21^

For immunoblotting, samples were separated by SDS-polyacrylamide gel electrophoresis under reducing conditions using a Tris-Glycine gel system, transferred to polyvinylidene difluoride membrane (Merck), and reacted with antibodies. Immunoblots were developed using ImmunoStar reagents (FUJIFILM Wako) or SuperSignal West Femto (Thermo Fisher Scientific) and visualized using LAS-4000 mini (Cytiva). A polyclonal IgG-HRP secondary antibody (NA931, GE healthcare), which recognizes both immunoglobulin heavy (IgH) and light (IgL) chains, was used for immunoblotting.

Aβ concentration in mouse or human brain extracts was quantified using a Human/Rat Aβ40 or Aβ42-specific ELISA (FUJIFILM Wako) as previously described.^22^ To measure Aβ oligomer levels, two Aβ oligomer-specific ELISA kits were used: High Molecular Amyloid-β Oligomer ELISA Kit (BAN50-BAN50, FUJIFILM Wako) and Human Amyloid-β oligomers Assay Kit (82E1-82E1, IBL).

### Protein extraction and size-exclusion chromatography

Extraction of frozen brains was described previously.^22^ Briefly, frozen human autopsied brains or mouse brain hemispheres were homogenized in a 1:10 (*w*/*v*) volume of Tris-buffered saline (TBS) with a cOmplete protease inhibitor cocktail (Merck) (TBSI), centrifuged at 267 000 *×g* for 20 min at 4°C, and the supernatants were used as TBS-soluble fraction. The resulting pellets were homogenized in a stepwise extraction with 1:10 (w/v) volume of 2% Triton X-100 in TBSI, a 1:10 (w/v) volume of 2% SDS in TBSI, 70% formic acid, and formic-acid-supernatants were desiccated with Speed-Vac followed by resuspension in dimethyl sulfoxide, and used as TBS-insoluble fractions. For repeated extractions, the resulting pellets of TBS-soluble fractions were re-homogenized in a 1:10 (*w*/*v*) volume of TBSI and centrifuged at 267 000 × *g* for 20 min at 4°C, four times. For negative staining electron microscopy, TBS-insoluble fractions were re-homogenized in extraction buffer (10 mM Tris-HCl with 0.8 M NaCl, 10% sucrose and 1 mM EGTA, pH = 7.5). Following a sonication, they were incubated with extraction buffer with 4% sarcosyl for 60 min at 37°C. Following a 5 min centrifugation at 3000 × *g*, the supernatants were centrifuged at 200 000 × *g* for 60 min. The pellets were re-homogenized in PBS and used for negative staining electron microscopy.

Brain TBS-soluble fractions were separated by size-exclusion chromatography (SEC) using Superdex75 10/300 GL (Cytiva) or Superdex75 10/300 GL increase (Cytiva) in 50 mM ammonium acetate (for Superdex75 10/300 GL) or PBS (for Superdex75 10/300 GL increase) at a flow rate of 0.5 mL/min using an AKTA Purifier 10 (Cytiva). For analyses of TBS-soluble Aβ oligomers in the brains of plaque-bearing APP tg mice, two Superdex75 10/300 GL columns connected in tandem were used, whereas a single Superdex75 10/300 GL increase column was used for analyses of TBS-soluble Aβ oligomers in the brains of patients with Alzheimer’s Disease and for the separation of Peak 1 Aβ from the brains of APP tg mice. Seven hundred microlitres of each brain TBS-soluble fraction was loaded onto an SEC column. After separation, samples were collected in 500 μL aliquots and analysed by ELISA and immunoblotting. Estimated molecular weight was determined based on the retention times of standard proteins from Gel Filtration Calibration Kits (LMW, HMW; Cytiva) under identical conditions.

### Immunodepletion and formic acid denaturation

For immunodepletion, 250 μL of Peak 1 Aβ fractions obtained from APP tg mouse brains was incubated with 30 μL of recombinant Protein G agarose beads (Thermo Fisher Scientific). After centrifugation at 8000 rpm for 5 min at 4°C, the supernatant was incubated with 5 μg of indicated antibodies for 12 h at 4°C, further incubated with 30 μL recombinant Protein G agarose beads for 2 h at 4°C and centrifuged to collect the supernatant. The same procedure was repeated four times, and the supernatant after immunodepletion was used for the *in vivo* seeding experiments.

For formic acid denaturation, Peak 1 Aβ fractions were incubated with 70% formic acid for 12 h at 4°C and dialysed against 3 changes of 1 L of PBS. The denatured sample was used for the *in vivo* seeding experiments.

### *In vivo* seeding experiments

**Figure 1:**
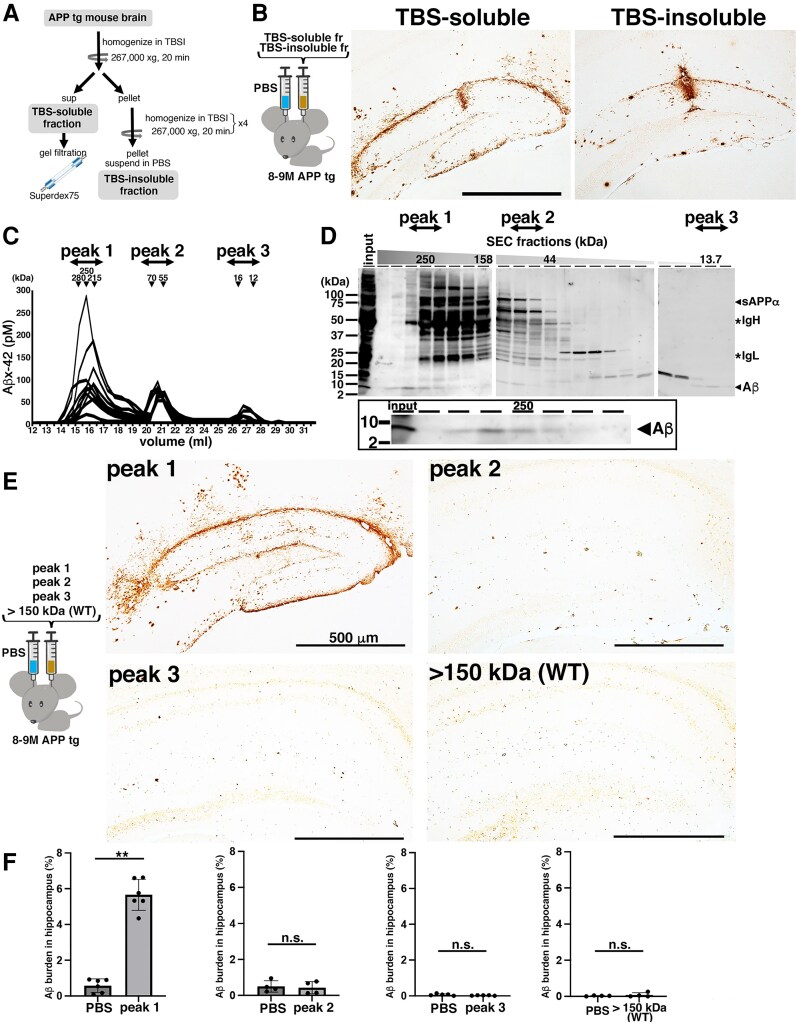
**Peak 1 amyloid-β (Aβ) from plaque-laden Aβ precursor protein transgenic (APP tg) mouse induces cerebral β-amyloidosis.** (**A**) Schematic representation of the method of extraction of Tris-buffered saline (TBS)-soluble and TBS-insoluble fractions from the brains of APP tg mice. (**B**) Immunohistochemical analysis of the brains of APP tg mice injected with TBS-soluble fraction (left) or TBS-insoluble fraction (right) into the hippocampus. Input represents TBS-soluble brain lysate prior to size-exclusion chromatography (SEC) separation. Scale bar shows 500 μm. (**C**) Separation of TBS-soluble fraction from brains of ten 18-month-old APP tg mice by SEC. Estimated molecular weight (kDa) is indicated at the top (arrowheads). The concentration of Aβx-42 was measured by enzyme-linked immunosorbent assay (ELISA) using the BNT77-BC05 Aβx-42-specific ELISA. Aβ was eluted into three different peaks, Peak 1 (>150 kDa), Peak 2 (50–70 kDa) and Peak 3 (10–20 kDa). (**D**) Representative immunoblotting of SEC-separated fractions using anti-Aβ monoclonal antibodies 82E1 and 6E10. Inset is enlarged image of Peak 1 fractions from 10 to 2 kDa. Approximately 4 kDa bands represent monomeric Aβ (arrowhead), and ∼80 kDa bands represent soluble APPα (sAPPα) (arrowhead). Approximately 25 and ∼50 kDa bands represent immunoglobulin light chain (IgL) and heavy chain (IgH), respectively (asterisks). The monomeric Aβ bands were detected in the Peak 1 and Peak 3 fractions. (**E**) Immunohistochemical analysis of brains of APP tg mice injected with Peak 1 (top left), Peak 2 (top right), Peak 3 (bottom left) derived from the brains of 18, 25, 26, 28, 29-month-old APP tg mice, or the >150 kDa SEC fraction from the brain of a 25-month-old wild-type mouse (bottom right). Representative images of Aβ staining show that the Peak 1 fraction induced Aβ deposition in the hippocampus, whereas Peak 2 and Peak 3 fractions, as well as >150 kDa SEC fraction from wild-type mice, did not. Scale bar shows 500 μm. (**F**) Morphometric analyses of amyloid burden (% Aβ-immunoreactive area) in the hippocampus of APP tg mice injected with Peak 1, Peak 2, Peak 3, or >150 kDa SEC fraction from a wild-type mouse (WT). *n* = 6 (Peak 1), 4 (Peak 2), 5 (Peak 3), 4 (>150 kDa fraction from WT). Each data point represents the mean area of Aβ burden in hippocampus quantified from five sections obtained from a single mouse. Contralateral hippocampi were injected with phosphate-buffered saline (PBS). Student’s *t*-test, ** *P* < 0.01, n.s., not significant.

*In vivo* seeding experiments for intrahippocampal injection were performed as previously described.^20^ Briefly, APP tg mice or wild-type mice were anaesthetized with chloral hydrate (400 mg/kg) and injected with 2.5 µL of samples into the hippocampus (anterior-posterior −2.5 mm, medial-lateral ±2.0 mm, dorsal −1.8 mm from bregma) using a 10 µL Hamilton syringe at a rate of 0.35 µL/min. In the comparison of seeding capacity per concentration of Aβ among the Peak 1, Peak 2 and Peak 3 fractions, Aβ42 concentration in each SEC fraction were determined by an Aβ42-specific ELISA. For this experiment, all samples were adjusted to 100 pM Aβ42 by dilution with PBS prior to injection (Fig. 1); in all subsequent experiments, SEC fractions were administered without dilution. Mice were anaesthetized and killed by decapitation at different predetermined time points [2, 4 or 6 months post-injection (m.p.i.)].

*In vivo* seeding experiments for intra-cisterna magna injection were performed with reference to a previous study.^23^ Four-month-old A7 mice were anaesthetized with a combination anaesthetic of 0.75 mg/kg medetomidine, 4.0 mg/kg midazolam and 5.0 mg/kg butorphanol. The muscle layers were bluntly dissected, and the dura mater overlaying the cisterna magna was exposed. A 5 µL of mice brain extracts A7 mice were injected into the subarachnoid space at a rate of 1 µg/min using a Hamilton syringe. After injection, the syringe was left in place for an additional 30 min to prevent backflow of CSF. Mice were anaesthetized and killed by decapitation at various pre-determined time points (3 and 4.5 months).

### Immunohistochemistry and morphometry

Mouse brains were immediately fixed with PBS containing 4% paraformaldehyde for 24 h, dehydrated and embedded in paraffin. Serial coronal sections were cut at 4-μm thickness. Deparaffinized sections were treated by microwave (550 W) for 10 min in citrate buffer pH 6.0, followed by reaction with 100 μg/mL of proteinase K (Worthington) in TBS for 6 min at 37°C. After blocking by incubation with 10% calf serum in TBS, the sections were incubated with an anti-Aβ antibody 82E1 (1:1000) and then with a biotinylated anti-mouse IgG antibody (Vector Laboratories), followed by visualization by the avidin-biotin complex method (ABC elite, Vector Laboratories).

The percentage area covered by Aβ immunoreactivity in the hippocampus was measured using Image J software (NIH) as previously described.^24^ Aβ-positive areas exhibiting brown colouration at a level above the threshold were binarized against the negative area, and the percentage covered by positive areas was calculated. Five consecutive sections 100 µm apart at the level of the injection site of the hippocampus (AP-2.5 mm) were examined, and the mean level of Aβ burden was calculated per for each animal.

### Statistical analyses

Tests for statistical significance between groups were performed using Prism6 (GraphPad, La Jolla, CA). Paired *t*-test or Student’s *t*-test for parametric tests in experiments involving two groups and 2-way ANOVA test for parametric tests in experiments involving three groups were performed. Correlations were evaluated using Pearson’s correlation coefficient with a two-tailed test.

## Results

### Peak 1 Aβ derived from brain lysate of plaque-laden APP tg mouse induces cerebral β-amyloidosis

The intracerebral administration of a brain lysate derived from plaque-laden APP tg mice leads to the development of cerebral β-amyloidosis in the APP tg mice,^12,13^ whereas Aβ protofibrils or fibrils pre-formed from synthetic Aβ polypeptides *in vitro* induce no or minimal Aβ deposition even at concentrations that are 1000 times higher than those found in the brain lysate,^13,20^ indicating that *in vitro* aggregated Aβ preparations do not contain the Aβ conformers needed to efficiently induce cerebral β-amyloidosis *in vivo*. To identify the specific Aβ species present in the brain lysate of plaque-laden APP tg mice that are involved in Aβ deposition, we extracted the TBS-soluble and insoluble fractions from the brains of plaque-laden 18-month-old APP tg mice (A7 line^11^) and stereotactically injected them at ∼1 nM concentration of Aβx-42 into the hippocampus of plaque-free 8- to 9-month-old APP tg mice (A7 line APP tg mice develop progressive Aβ deposition in the brain starting at 10–11 months of age). Four months later, immunohistochemical analysis revealed that TBS-soluble fraction induced Aβ deposition with a unique laminar pattern along the hippocampal dentate gyrus (Fig. 1A and B), which was similar to the previous studies.^13,20^ In contrast, Aβ deposition was observed around the injection site in the hippocampus of APP tg mice inoculated with the TBS-insoluble fraction (Fig. 1B). Based on these findings, we postulated that the Aβ species responsible for Aβ deposition and its spatiotemporal spreading are more abundant in the TBS-soluble fraction than in the TBS-insoluble fraction.

In order to identify the specific Aβ species involved in the induction of Aβ deposition, we separated the TBS-soluble fraction of APP tg mice using SEC with a Superdex75 column and quantified the concentration of Aβ using an Aβ-specific ELISA. We discovered that Aβ species within the TBS-soluble fraction of plaque-laden 18- to 19-month-old APP tg mice eluted into three fractions, with molecular weights of >150 kDa, 50–70 kDa and 10–20 kDa, hereinafter referred to as Peak 1, Peak 2 and Peak 3, respectively (Fig. 1C). Immunoblotting of SEC-separated fractions from 18- to 19-month-old APP tg mice revealed a band of approximately 4 kDa corresponding to Aβ monomer in Peak 1 and Peak 3 fractions; however, no bands corresponding to monomeric Aβ were detected in the Peak 2 fractions (Fig. 1D).

To compare the ability of Peak 1, Peak 2 and Peak 3 Aβ to induce Aβ deposition, we stereotactically injected each fraction normalized by Aβ concentration into the hippocampus of plaque-free APP tg mice. Four months later, immunohistochemical analysis revealed that Peak 1 Aβ induced a characteristic band-shaped Aβ deposition in the hippocampus, whereas no Aβ deposition was observed in the hippocampus of APP tg mice injected with Peak 2 Aβ or Peak 3 Aβ (Fig. 1E). The average area occupied by Aβ in the hippocampus of mice injected with Peak 1 Aβ was ∼6.0%, which was ∼12-fold higher than that in the contralateral hippocampus of mice injected with PBS (Fig. 1F). Additionally, no Aβ deposition was observed in the hippocampus of APP tg mice injected with a >150 kDa fraction (equivalent molecular weight to Peak 1 Aβ) extracted from the TBS-soluble fraction of 18-month-old wild-type mice (Fig. 1E and F). Upon closer observation, Aβ was found to be deposited along the molecular layer or the polymorphic layer (i.e. a.k.a. hilus or CA4) of the hippocampal dentate gyrus, along the hippocampal fissure, or beneath the pial surface (Supplementary Fig. 1). Additionally, Aβ was abundantly deposited at the stratum lacunosum-moleculare of the hippocampus (Supplementary Fig. 1). Notably, this distribution of Aβ deposits resembles that described previously in the medial temporal lobe.^4^

To rule out the possibility that Peak 1 Aβ is just an artefact resulting from the break-down of insoluble Aβ fibrils during the brain homogenization process, we extracted the same brains from 18- to 19-month-old APP tg mice repeatedly with up to five rounds of homogenization in TBS followed by ultracentrifugation and subjected each of the five resulting TBS-soluble fractions to SEC separation. Peak 1 Aβ was detected only in the first TBS-soluble sample, suggesting that Peak 1 Aβ is not just the result of mechanical disruption of insoluble Aβ fibrils from plaques (Supplementary Fig. 2).

In order to explore the potential presence of amyloid fibrils in the Peak 1 fraction, the Peak 1 or TBS-insoluble fraction obtained from brains of 18- to 19-month-old APP tg mice was analysed by negative staining electron microscopy. In the TBS-insoluble fraction, we observed linear amyloid fibrils with >100 nm in length; however, no amyloid fibrils were observed in the Peak 1 fraction, suggesting that amyloid fibrils might not constitute the principal substance responsible for the seeding ability of Peak 1 Aβ (Supplementary Fig. 3).

To investigate the Aβ species deposited in the hippocampus of APP tg mice following Peak 1 Aβ injection, we conducted immunohistochemical analyses utilizing carboxy-terminal-specific antibodies for Aβ40, Aβ42 or Aβ43. Our results indicated a predominant presence of Aβ42 in the Aβ deposits, with minimal detection of Aβ40 or Aβ43 (Supplementary Fig. 4). Consequently, the levels of Aβ42 were evaluated in the brains of Peak 1 Aβ-injected mice using a specific ELISA. Because Aβ species deposited in senile plaques in the brains of patients with Alzheimer’s disease are known to be frequently amino-terminally truncated and modified at position 3 by pyroglutamate formation,^25,26^ we further performed immunohistochemical analysis using an antibody specific for pyroglutamate-modified Aβ at position 3 (N3pE). However, no detectable signals were observed in the induced Aβ deposits, suggesting that pyroglutamate-modified Aβ contributes minimally to the deposited Aβ species (Supplementary Fig. 5).

**Figure 2:**
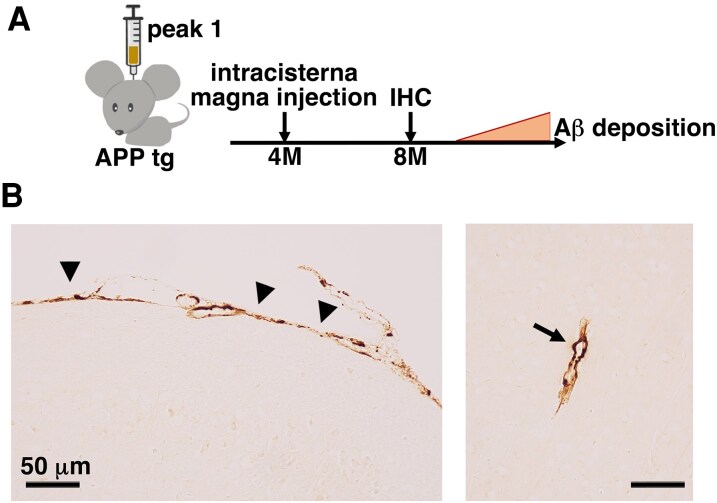
**Peak 1 amyloid-β (Aβ) induces cerebral amyloid angiopathy.** (**A**) Schematic representation of the method of intracisternal injection of Peak 1 Aβ via cisterna magna of 4-month-old Aβ precursor protein transgenic (APP tg) mice. (**B**) Immunohistochemical analyses of brains of APP tg mice injected Peak 1 Aβ derived from the brains of 25-month-old APP tg mice. Scale bar indicates 50 μm. Arrowheads indicate Aβ deposits within the wall of leptomeningeal arteries, and an arrow indicates cerebral amyloid angiopathy. IHC, immunohistochemistry.

We further administered Peak 1 Aβ directly into the cerebrospinal fluid of plaque-free APP tg mice via the cisterna magna. We found that Peak 1 Aβ predominantly induced Aβ deposition within the wall of leptomeningeal arteries surrounding the brain, reminiscent of cerebral amyloid angiopathy (CAA) (Fig. 2). This observation suggested that Peak 1 Aβ may not only induce amyloid plaques but also contribute to CAA formation. Together, these data support the hypothesis that Peak 1 Aβ exhibits seeding capability for β-amyloidosis in the brain.

### Temporal accrual of Peak 1 Aβ in APP tg mouse brains

**Figure 3:**
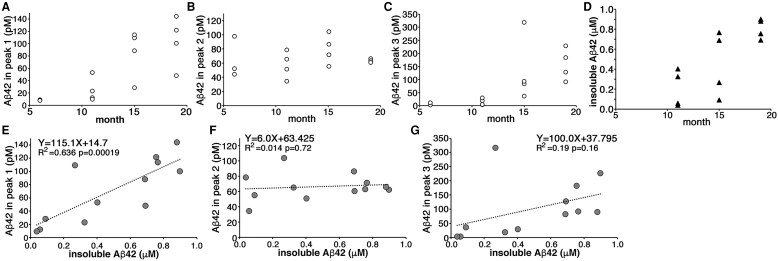
**Temporal accrual of Peak 1 amyloid-β (Aβ) in the Aβ precursor protein transgenic (APP tg) mouse brain.** (**A–C**) Concentration of Aβx-42 in Peak 1 (**A**, 8.1 ± 0.73, 24.4 ± 19.9, 84.9 ± 39.2 and 103.5 ± 41.0 pM at 6, 11, 15 and 18–19 months, respectively), Peak 2 (**B**, 64.6 ± 28.8, 57.4 ± 19.0, 79.2 ± 20.8 and 63.0 ± 2.3 pM at 6, 11, 15 and 18–19 months, respectively) or Peak 3 fraction (**C**, 5.9 ± 4.4, 13.7 ± 12.6, 131.8 ± 125.3 and 148.0 ± 67.8 pM at 6, 11, 15 and 18–19 months, respectively) in the brains of 6-, 11-, 15- and 18- to 19-month-old APP tg mice. *n* = 3 (6-month-old) or *n* = 4 (11, 15 and 18- to 19-month-old). (**D**) Concentration of Aβ42 in the brains of 11-, 15- and 18- to 19-month-old APP tg mice *n* = 4. 0.21 ± 0.12, 0.45 ± 0.33 and 0.81 ± 0.10 μM/g wet tissue at 11, 15 and 18–19 months, respectively. (**E–G**) Correlation between concentration of Aβx-42 in Peak 1 (**E**, *R^2^* = 0.636, *P* = 0.00019), Peak 2 (**F**, *R^2^* = 0.014, *P* = 0.72) or Peak 3 (**G**, *R^2^* = 0.19, *P* = 0.16) fraction and insoluble Aβ42. Correlations were evaluated using Pearson’s correlation coefficient with a two-tailed test. Each data point represents an individual mouse. (**A–D**) Show the concentration of Aβx-42 in each peak fraction, whereas (**E–G**) show the relationship between these values and insoluble Aβ levels.

To determine the temporal pattern of accumulation of TBS-soluble Peak 1, Peak 2 and Peak 3 Aβ within the brain relative to insoluble Aβ, we quantified the amount of Aβx-42 in Peak 1, Peak 2 and Peak 3, as well as the amount of insoluble Aβx-42 (SDS-insoluble/70% formic acid-soluble) in the brains of 6-, 11-, 15- and 18- to 19-month-old APP tg mice via ELISA. We found that the levels of Peak 1 Aβ increased with age in parallel to the levels of insoluble Aβ (Fig. 3A and D). Indeed, we found a strong positive correlation between the levels of Peak 1 Aβ and the levels of insoluble Aβ (Fig. 3E). We also found a similar age-associated increase in the concentration of Peak 3 Aβ (Fig. 3C). In contrast, the levels of Peak 2 Aβ did not differ between 6 and 19 months (Fig. 3B). We did not see any positive correlation between the levels of Peak 2 or Peak 3 Aβ and the levels of insoluble Aβ (Fig. 3F and G).

### Spatiotemporal spreading of β-amyloidosis induced by Peak 1 Aβ in the brain

**Figure 4:**
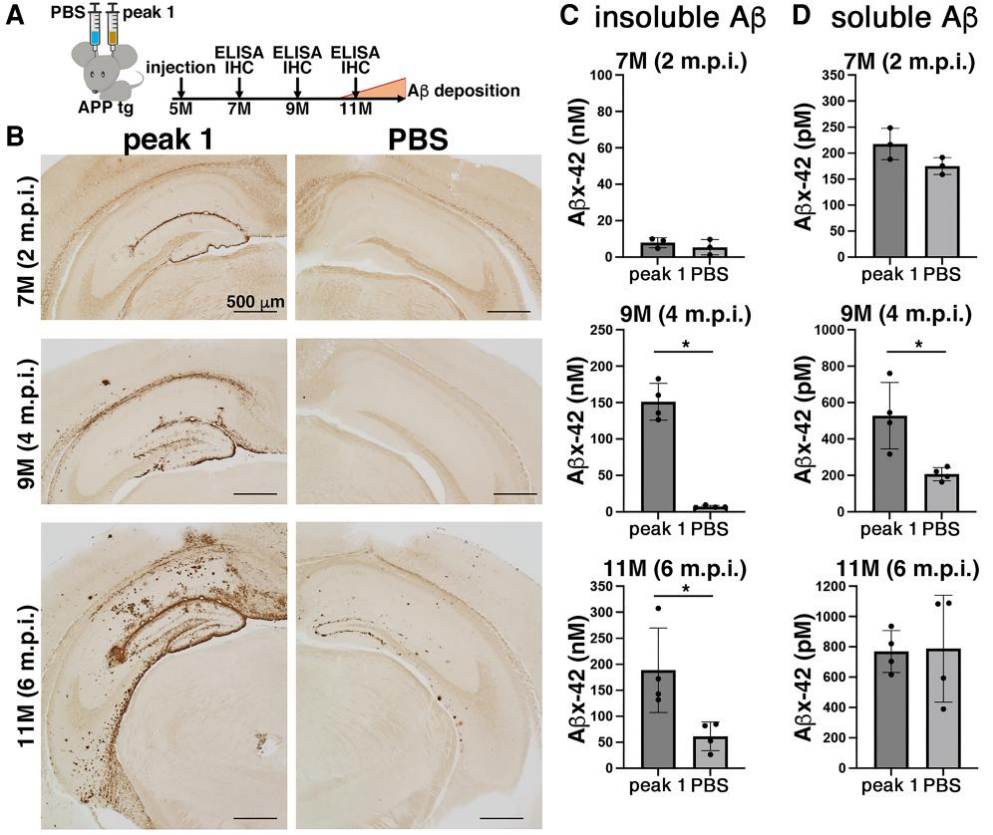
**Spatiotemporal spreading of β-amyloidosis induced by Peak 1 amyloid-β (Aβ) in the brain.** (**A**) Time course of *in vivo* seeding experiments, immunohistochemical analyses and enzyme-linked immunosorbent assays. (**B**) Representative images of immunohistochemical staining of 7-, 9- or 11-month-old Aβ precursor protein transgenic (APP tg) mice injected with Peak 1 Aβ derived from the brains of 25, or 30-month-old APP tg mice (left) or phosphate-buffered saline (PBS) (right) using an anti-Aβ antibody (82E1). Scale bar shows 500 μm. (**C**, **D**) Concentration of Aβx-42 in the sodium dodecyl sulphate-insoluble/formic acid-soluble fraction (insoluble Aβ, C, 7.9 ± 1.6 nM in Peak 1 Aβ side 2 months post-injection (m.p.i.), 5.4 ± 2.4 nM in PBS side 2 m.p.i., 151.2 ± 12.6 nM in Peak 1 Aβ side 4 m.p.i., 7.1 ± 0.8 nM in PBS side 4 m.p.i.188.4 ± 40.6 nM in Peak 1 Aβ side 6 m.p.i., 61.4 ± 13.7 nM in PBS side 6 m.p.i.), or the Tris-buffered saline-soluble fraction (soluble Aβ, D, 217.6 ± 17.4 pM in Peak 1 Aβ side 2 m.p.i., 175.2 ± 9.4 pM in PBS side 2 m.p.i., 527.9 ± 91.4 pM in Peak 1 Aβ side 4 m.p.i., 206.8 ± 18.2 pM in PBS side 4 m.p.i., 769.1 ± 69.2 pM in Peak 1 Aβ side 6 m.p.i.788.3 ± 176.3 pM in PBS side 6 m.p.i.) in the brains of 7-, 9- or 11-month-old APP tg mice injected with Peak 1 Aβ or PBS. *n* = 3 for 7-month-old APP tg mice and *n* = 4 for 9- or 11-month-old APP tg mice, Student’s *t*-test, *P < 0.05. Each data point represents the insoluble or soluble Aβ concentration obtained from a single mouse. IHC, immunohistochemistry.

To delineate the spatiotemporal spreading of Aβ deposits, we injected Peak 1 Aβ into the ipsilateral hippocampus or PBS into the contralateral hippocampus of plaque-free 5-month-old APP tg mice and analysed their brains immunohistochemically and biochemically 2-, 4- and 6 m.p.i. corresponding to 7, 9 and 11 months of age (Fig. 4A). Immunohistochemical observation 2 m.p.i. revealed the presence of Aβ deposits along the molecular layer of the hippocampal dentate gyrus and the pial surface of the hippocampus in the Peak 1 Aβ-injected hemisphere. Aβ deposition had extended to the polymorphic layer of the dentate gyrus and the stratum lacunosum-moleculare of the hippocampus 4 m.p.i. (Figure 4B). Interestingly, abundant Aβ deposits were observed throughout the hippocampus in the injected hemisphere and, noteworthy, also in the molecular layer of the dentate gyrus of the contralateral hippocampus 6 m.p.i. (Figure 4B). Because A7 line exhibits very little Aβ deposits in hippocampus at 11 to 12 months,^11^ Aβ deposits observed in the PBS-injected hippocampus 6.m.p.i. were thought to have propagated from the hippocampus injected with Peak 1 Aβ. We also analysed the extent of Aβ deposition in the anterior–posterior axis. Aβ deposits were observed in the posterior regions of the hippocampal dentate gyrus 4 m.p.i. (Supplementary Fig. 6). Additionally, we quantified the soluble (TBS-soluble) and insoluble (SDS-insoluble and formic-acid-soluble) Aβx-42 levels in the Peak 1 Aβ- and PBS-injected cortices. We found that the amount of insoluble Aβx-42 in the Peak 1 Aβ-injected hemisphere was significantly increased compared to the PBS-injected hemisphere 4 and 6 m.p.i. but not 2 m.p.i. (Figure 4C). Conversely, the amount of soluble Aβx-42 in the Peak 1 Aβ-injected hemisphere was significantly increased 4 m.p.i. compared with the PBS-injection hemisphere, but no difference was observed between them 2 or 6 m.p.i. (Figure 4D). These results suggest that β-amyloidosis induced by Peak 1 Aβ spreads through the brain.

### Aβ oligomers are essential for the induction of β-amyloidosis by Peak 1 aβ

**Figure 5:**
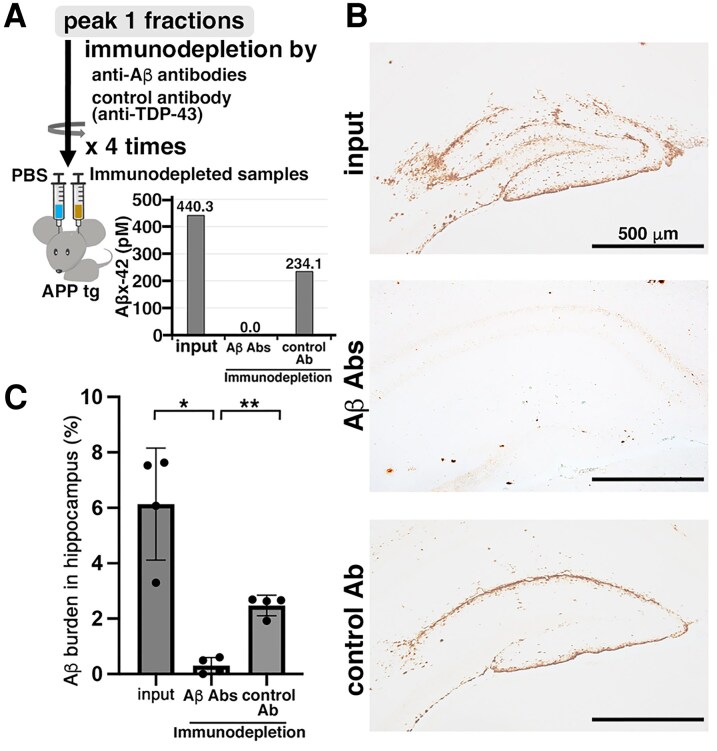
**Amyloid-β (Aβ) is essential for the induction of β-amyloidosis by Peak 1 Aβ.** (**A**) Schematic representation of the method of the immunodepletion and *in vivo* seeding experiment. The concentration of Aβx-42 (pM) in Peak 1 Aβ before and after immunodepletion with anti-Aβ antibodies BAN50 and 4G8 or control antibody was measured by specific enzyme-linked immunosorbent assay and presented as a bar graph. Each bar represents Aβx-42 concentration in an independent sample used for *in vivo* seeding experiments (*n* = 1). (**B**) Representative images of immunohistochemical staining of Aβ precursor protein transgenic (APP tg) mice injected with Peak 1 Aβ (input, top), Peak 1 Aβ immunodepleted with anti-Aβ antibodies (middle), or Peak 1 Aβ immunodepleted with control antibody (bottom). The scale bar shows 500 μm. (**C**) Morphometric analyses of the amyloid burden (% Aβ−immunoreactive area) in the hippocampus of APP tg mice injected with Peak 1 Aβ, Peak 1 Aβ immunodepleted with anti-Aβ antibodies or Peak 1 Aβ immunodepleted with control antibody. *n* = 4, Student’s *t*-test, * *P* < 0.05, ** *P* < 0.01. Each data point represents the mean Aβ burden in hippocampus quantified from five sections obtained from a single mouse. Ab, antibody.

We hypothesized that the presence of Aβ is essential for the induction of β-amyloidosis by Peak 1 Aβ. To test this, we carried out the immunodepletion of Aβ from Peak 1 Aβ using anti-Aβ monoclonal antibodies BAN50 and 4G8 and compared the seeding effects of Aβ-immunodepleted and non-immunodepleted Peak 1 *in vivo*. We achieved an almost complete elimination of Aβ, whereas the concentration of Aβx-42 in the Peak 1 Aβ immunodepleted with a control anti-TDP-43 antibody was ∼234.1 pM (Fig. 5A). In the *in vivo* seeding experiment, we found no Aβ deposits 4 months after injection of Aβ-immunodepleted Peak 1 into the hippocampus of 7-month-old APP tg mice (Fig. 5B and C). In contrast, non-immunodepleted Peak 1 Aβ and Peak 1 Aβ immunodepleted with a control antibody led to the characteristic band-shaped Aβ deposition in the hippocampus (Fig. 5B and and C). These results suggest that Aβ species contained in Peak 1 Aβ are essential for the induction of Aβ deposition.

**Figure 6:**
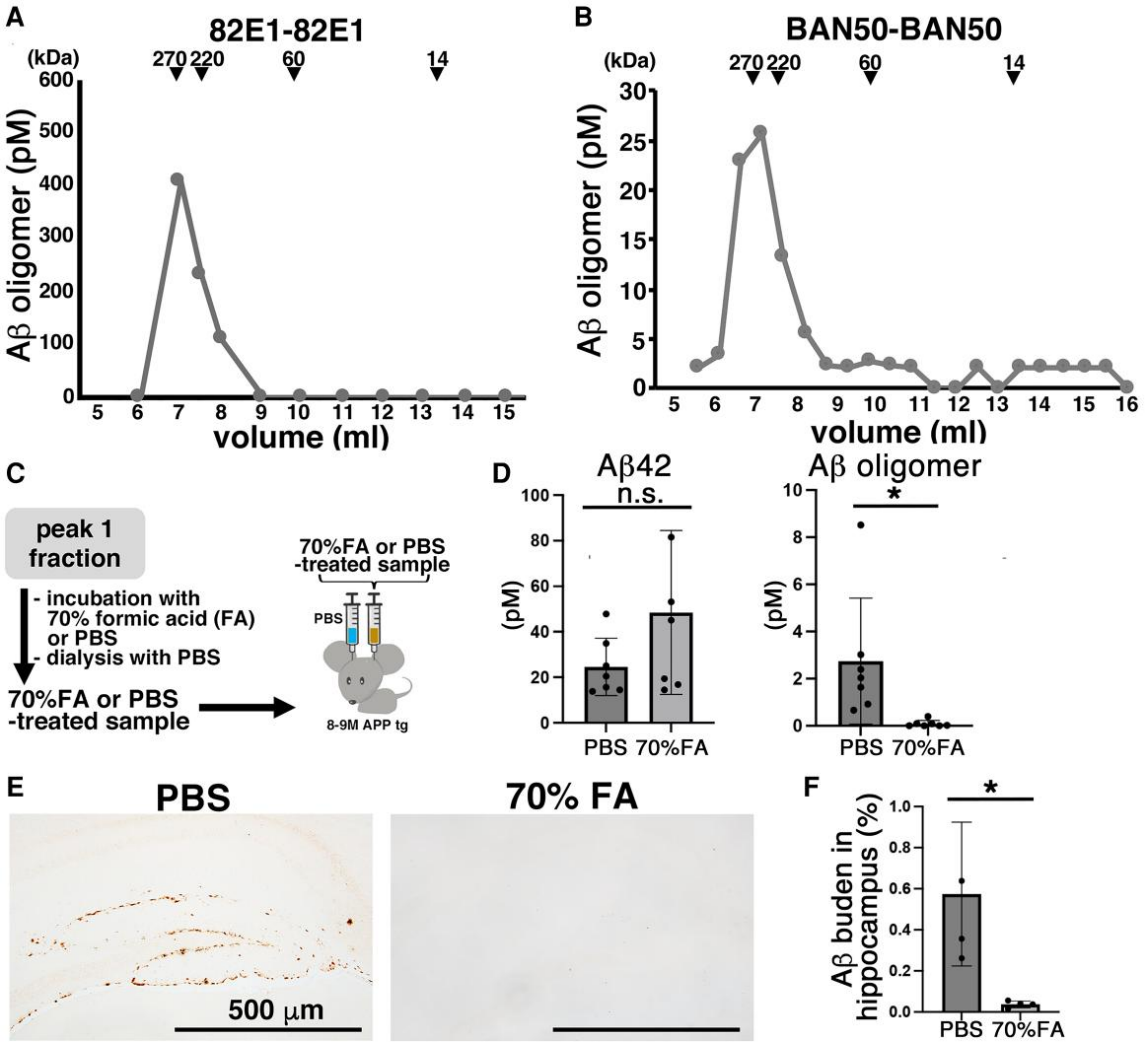
**Amyloid-β (Aβ) oligomers in Peak 1 Aβ are essential for the induction of β-amyloidosis.** (**A**, **B**) Representative data of Aβ oligomer concentration (pM) in size-exclusion chromatography (SEC)-separated fractions using the Aβ oligomer-specific single-antibody sandwich enzyme-linked immunosorbent assay (ELISA) 82E1-82E1 (**A**) or BAN50-BAN50 (**B**). Estimated molecular weight (kDa) is indicated at the top (arrowheads). Each data point represents the Aβ oligomer level measured by ELISA in each SEC fraction from an individual mouse, with each mouse representing an independent biological replicate (*n* = 3). (**C**) Schematic representation of the procedure of formic acid denaturation and *in vivo* seeding. (**D**) The concentration of Aβx-42 (left) or Aβ oligomers (right) in Peak 1 Aβ derived from the brains of 29-month-old Aβ precursor protein transgenic (APP tg) mice treated with 70% formic acid (FA) or phosphate-buffered saline (PBS). *n* = 7, paired *t*-test, * *P* < 0.05, n.s., not significant. Each data point represents the Aβx-42 or Aβ oligomer concentration obtained from a single mouse. (**E**) Representative images of immunohistochemical staining of APP tg mice injected with Peak 1 Aβ pre-treated with PBS (left) or 70% FA (right) using anti-Aβ antibody (82E1). Representative images show that Aβ deposition is observed in PBS-treated samples, whereas no Aβ deposition is detected in FA-treated samples. Scale bar shows 500 μm. (**F**) Morphometric analyses of the amyloid burden (% Aβ-immunoreactive area) in the hippocampus of APP tg mice pre-treated with PBS or 70% FA. *n* = 4, paired *t*-test, * *P* < 0.05. Each data point represents the mean Aβ burden in hippocampus quantified from five sections obtained from a single mouse.

It has been reported that Aβ forms soluble oligomers *in vitro* as well as in the brains of patients with Alzheimer’s disease and APP tg mice and that these Aβ oligomers are involved in Aβ fibrillization and deposition and synaptic dysfunction.^27-36^ We first investigated whether Aβ oligomers are present in Peak 1 Aβ using a single-site sandwich ELISA to specifically measure Aβ oligomers.^37,38^ TBS-soluble fractions of 22-month-old APP tg mouse brains were separated by SEC and level of Aβ oligomers were measured by 82E1-82E1 or BAN50-BAN50 Aβ oligomer-specific ELISA. We found that Aβ oligomers were detected in the Peak 1 Aβ fractions but not in the Peak 2 or 3 Aβ fractions (Fig. 6A and B). To further investigate whether Aβ oligomers in Peak 1 Aβ are involved in the induction of β-amyloidosis, Peak 1 Aβ was incubated with 70% formic acid, which is known to denature the β-sheet structures of amyloid fibrils,^13,39,40^ followed by dialysis with PBS. Incubation with 70% formic acid resulted in the loss of Aβ oligomers in Peak 1 Aβ, whereas the level of Aβx-42 in Peak 1 Aβ did not change (Fig. 6D). We then injected 70% formic acid pre-treated Peak 1 Aβ versus PBS-treated Peak 1 Aβ into the hippocampus of 8- to 9-month-old APP tg mice. Four months later, we found that 70% formic acid pre-treated Peak 1 Aβ failed to induce deposition in the hippocampus, whereas PBS-treated Peak 1 Aβ induced Aβ deposition (Fig. 6C and E, 6F). Thus, these results indicate that the presence of Aβ oligomers in Peak 1 Aβ is indispensable for the induction of β-amyloidosis.

### β-amyloidosis induced by Peak 1 Aβ caused accumulation of astrocytes and microglia

**Figure 7:**
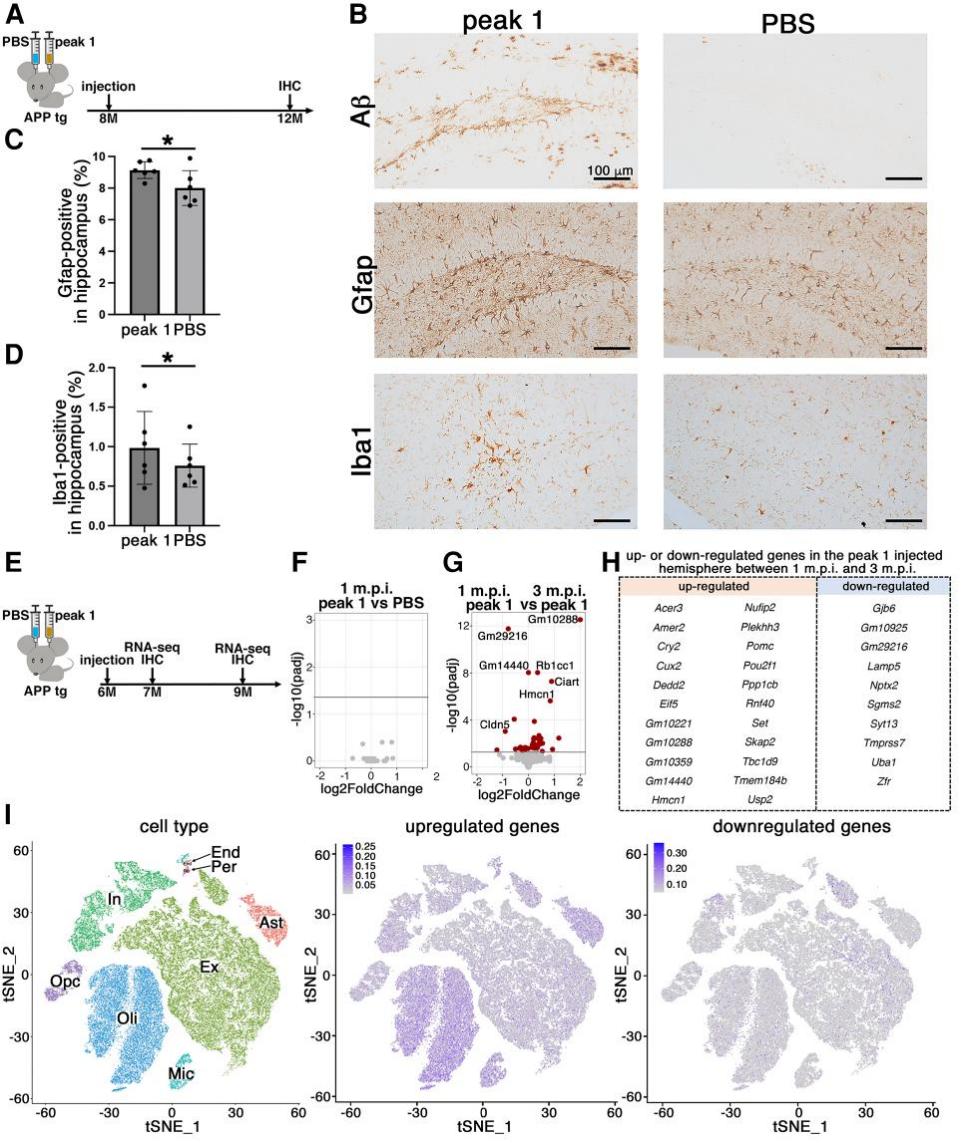
**β-amyloidosis induced by Peak 1 amyloid-β (Aβ) elicits reactive gliosis in the brain.** (**A**) Time course of *in vivo* seeding experiments and immunohistochemical analyses. (**B**) Representative images of immunohistochemical staining of Aβ precursor protein transgenic (APP tg) mice injected with Peak 1 Aβ derived from the brains of 29-month-old APP tg mice or phosphate-buffered saline (PBS) using anti-Aβ antibody (top), anti-glial fibrillary acidic protein (Gfap) antibody (middle), or anti-Iba1 antibody (bottom). Representative images show that Gfap-positive astrocytes or Iba1-positive microglia accumulate around Aβ deposits induced by the Peak 1 Aβ, whereas such accumulation is absent in PBS-treated samples. (**C**, **D**) Relative immunoreactive areas (%) of anti-Gfap (**C**) or anti-Iba1 (**D**) antibody in the hippocampus of APP tg mice injected with Peak 1 Aβ or PBS. *n* = 6, paired *t*-test, * *P* < 0.05. Each data point represents the anti-Gfap- or anti-Iba1-positive area in hippocampus quantified from five sections obtained from a single mouse. (**E**) Time course of *in vivo* seeding experiments, immunohistochemical analyses and RNA-sequencing (RNA-seq). (**F**) RNA-Seq volcano plot of differential gene expression analysis in the brain hemispheres injected with Peak 1 Aβ compared to the brain hemispheres injected with PBS after 1 month. The horizontal grey line corresponds to a significance value of 0.05. The horizontal grey line corresponds to an FDR-adjusted *P*-value of 0.05 (Benjamini–Hochberg correction). Total transcripts, *n* = 14 819. No transcripts met the significance threshold. (**G**, **H**) RNA-Seq volcano plot of differential gene expression analysis in the brain hemispheres injected with Peak 1 Aβ 1 (months post-injection) m.p.i. versus 3 m.p.i. The horizontal grey line corresponds to an FDR-adjusted *P*-value of 0.05 (Benjamini–Hochberg correction). Total transcripts, *n* = 14 687. A total of 32 transcripts met the significance threshold. Statistically significant up- or down-regulated genes are listed in **H**. (**I**) Two-dimensional t-stochastic neighbour embedding (tSNE) visualization of brain single-nucleus RNA-seq (snRNA-seq) data from Mathys *et al.*^25^ (left). Upregulated genes (middle) or downregulated genes (right) in the brains of APP tg mice injected with Peak 1 Aβ were mapped on the tSNE plot to identify main brain cell types implicated. tSNE plot of snRNA-seq data (total nuclei, *n* = 70 634). Clusters are annotated as astrocytes (Ast, *n* = 3392), endothelial cells (End, *n* = 121), excitatory neurons (Ex, *n* = 34 976), inhibitory neurons (In, *n* = 9196), microglia (Mic, *n* = 1920), oligodendrocytes (Oli, *n* = 18 235), oligodendrocyte precursor cells (Opc, *n* = 2627) and pericytes (Per, *n* = 167). IHC, immunohistochemistry.

The association of reactive astrocytes and microglia around and within the senile plaques is a characteristic feature in the brains of patients with Alzheimer’s disease.^41^ To investigate whether β-amyloidosis induced by Peak 1 Aβ stimulates the reaction of astrocytes or microglia, we injected stereotactically Peak 1 Aβ into the hippocampus of 8- to 9-month-old APP tg mice and performed immunohistochemical analyses 4 m.p.i. using an anti-Aβ antibody, an anti-Gfap antibody for astrocytes and an anti-Iba1 antibody for microglia (Fig. 7A). We found reactive astrocytes and microglia associated with the Aβ deposits induced by Peak 1 Aβ mainly in the dentate gyrus of the hippocampus (Fig. 7B), resulting in a significantly increased average positive-area immunostained by anti-Gfap and anti-Iba1 antibody in the Peak 1 Aβ-injected hippocampus compared with the PBS-injected hippocampus (Fig. 7C and D). To elucidate the molecular mechanisms underlying these astrocytic and microglial responses associated with the Peak 1 Aβ-induced β-amyloidosis, we performed bulk RNA-seq in the hemibrains of Peak 1 Aβ-injected mice 1 m.p.i. (i.e. when no Aβ deposition is observed), and 3 m.p.i. (i.e. when β-amyloidosis is already induced) (Fig. 7E). Differential gene expression analyses revealed no genes significantly changed in expression between the Peak 1 Aβ-injected and PBS-injected hemispheres 1 m.p.i. (Figure 7F). When comparing gene expression levels in Peak 1 Aβ-injected mice across 1 m.p.i. and 3 m.p.i., we found that the expression levels of 32 genes were significantly changed at 3 m.p.i. versus 1 m.p.i. (Figure 7G and H), of which 22 genes were upregulated and 10 genes were downregulated. To investigate which brain cell types were affected by the β-amyloidosis, we mapped these 32 genes onto a public snRNA-seq dataset from the dorsolateral prefrontal cortex of Alzheimer’s disease and control subjects^42^ and found that most of the upregulated genes are expressed by astrocytes, microglia, or oligodendrocytes, whereas most of the downregulated genes are expressed by excitatory or inhibitory neurons (Fig. 7I). We further investigated whether the expression levels of these 32 genes are altered in the brains of patients with Alzheimer’s disease along Braak stages.^42^ Our analysis revealed that the upregulated gene *TMEM184B* showed increased expression in astrocyte at the early stage, while *AMER2* exhibited increased expression in oligodendrocyte at the late stage, and *SKAP2* exhibited increase expression in microglia at the late stage (Supplementary Fig. 7). Taken together, these results suggest that β-amyloidosis induced by Peak 1 Aβ causes changes in gene expression in both neurons or glia, which may lead to reactive gliosis and local neurotoxicity.

### Peak 1 Aβ is present in the brains of patients with Alzheimer’s disease

**Figure 8:**
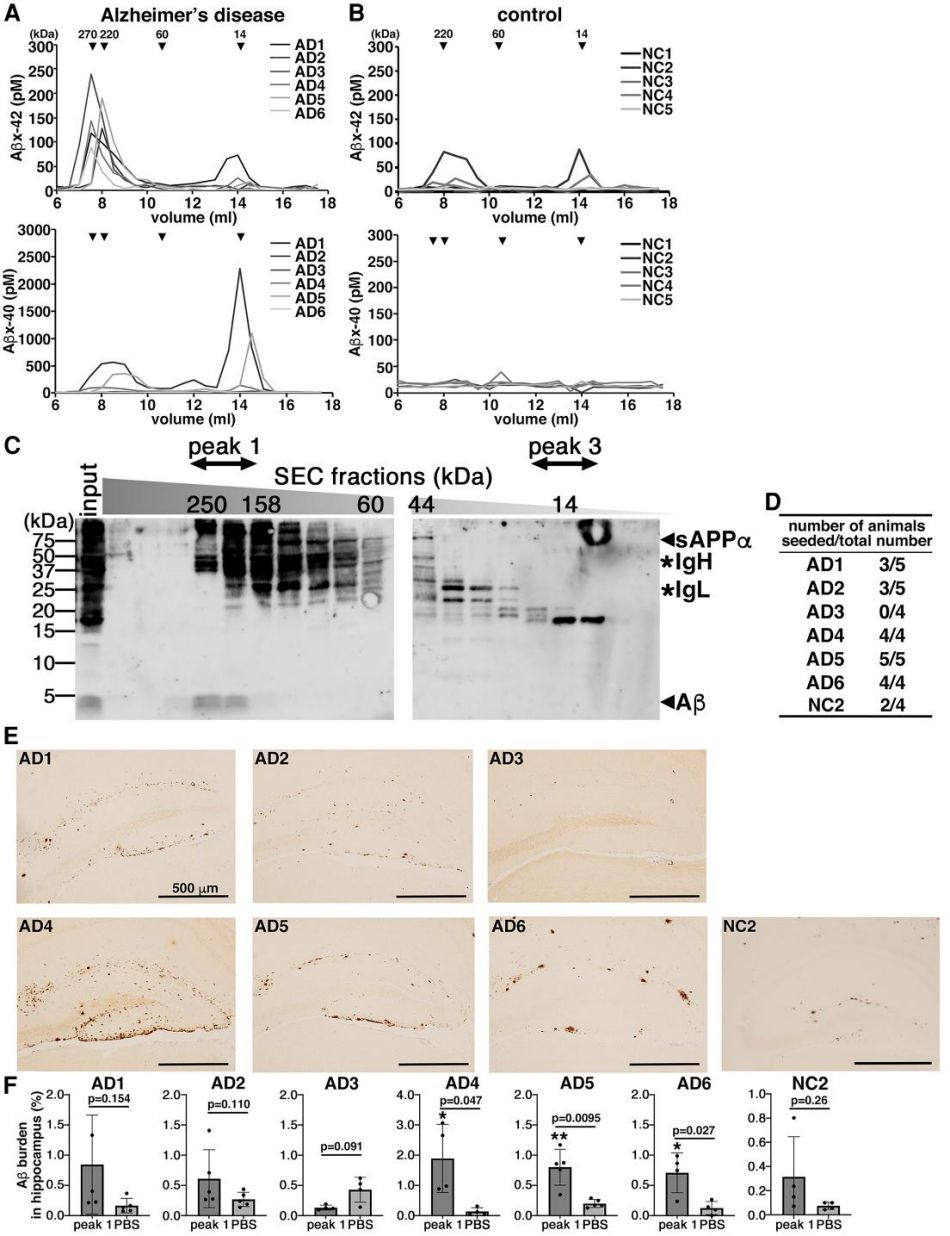
**Peak 1 amyloid-β (Aβ) is present in the brains of patients with Alzheimer’s disease.** (**A**, **B**) Separation of the Tris-buffered saline-soluble fraction from the brains of 6 Alzheimer’s disease donors (**A**) and 5 controls (**B**). Estimated molecular weight (kDa) is indicated at the top (arrowheads). The concentration of Aβx-42 (upper) or Aβx-40 (lower) was measured by enzyme-linked immunosorbent assay. (**C**) Representative immunoblotting of size-exclusion chromatography (SEC)-separated fractions using anti-Aβ monoclonal antibodies 82E1 and 6E10. Approximately 4 kDa bands represent monomeric Aβ (arrowhead), and ∼80 kDa bands represent soluble Aβ precursor protein α (sAPPα) (arrowhead). Approximately 25 and ∼50 kDa bands represent immunoglobulin light chain (IgL) and heavy chain (IgH), respectively (asterisks). The monomeric Aβ bands were detected in the Peak 1 fractions. (**D**) Number of mice with induced Aβ deposition in *in vivo* seeding experiments using Peak 1 Aβ from six patients with Alzheimer’s disease or one control (NC2). (**E**) Representative images of immunohistochemical staining of Aβ precursor protein transgenic (APP tg) mice injected with Peak 1 Aβ from six patients with Alzheimer’s disease or one control (NC2). (**F**) Morphometric analyses of the amyloid burden (% Aβ-immunoreactive area) in the hippocampus of APP tg mice injected with Peak 1 Aβ from six patients with Alzheimer’s disease or one control (NC2). *n* = 4–5, Student’s *t*-test, * *P* < 0.05, ** *P* < 0.01. Each data point represents the mean Aβ burden in hippocampus quantified from five sections obtained from a single mouse.

Finally, we examined whether the presence of Peak 1 Aβ is implicated in the deposition and spreading of Aβ in the brains of patients with Alzheimer’s disease. We analysed brain samples from six patients with Alzheimer’s disease (Braak stage V/VI, Supplementary Fig. 8A) and five control individuals without dementia (Braak stage I/II) with TBS-extraction, followed by separation via SEC, and measurement of Aβx-42 concentration in SEC fractions (Fig. 8A and B). Our findings revealed that >150 kDa Peak 1 Aβ was present in the brains of all patients with Alzheimer’s disease, as well as one elderly control brain. Conversely, the 50–70 kDa Peak 2 Aβ was not detected in any of the Alzheimer’s disease or control brains, while the 10–20 kDa Peak 3 Aβ was detected in the brains of five out of six Alzheimer’s disease donors and two out of five control brains (Fig. 8A and B). To further explore the levels of Aβ species in Peak 1 fractions, we quantified the levels of Aβx-40 in SEC fraction using Aβx-40-specific ELISA and found that the Peak 1 or Peak 3 fractions from AD1, AD3 or AD5 cases contained both Aβx-40 and Aβx-42, whereas minimal Aβx-40 was detected in the Peak 1 or Peak 3 fractions from AD2, AD4 or AD6 cases (Fig. 8A and B). Immunoblotting of SEC-separated samples revealed ∼4 kDa bands corresponding to Aβ monomer in Peak 1 fractions, whereas no Aβ monomer band was observed in the Peak 3 fractions (Fig. 8C). Interestingly, the ∼4 kDa Aβ bands in the Peak 1 fractions appeared as a double, suggesting that Peak 1 Aβ derived from Alzheimer’s disease patient brains may comprise multiple Aβ species. Further mass spectrometric analysis of Peak 1 Aβ from Alzheimer’s disease brains will be required to more clearly define the Aβ species involved.

To investigate whether Peak 1 Aβ from Alzheimer’s disease brains can induce β-amyloidosis, we stereotactically injected Peak 1 Aβ from Alzheimer’s disease brains into the hippocampus of 8- to 9-month-old APP tg mice (*n* = 4–5) and evaluated the presence of Aβ deposits 4 m.p.i. by immunohistochemistry with an anti-Aβ antibody. Our results revealed that Peak 1 Aβ from AD4, AD5 and AD6 donors induced Aβ deposition along the molecular layer of the hippocampal dentate gyrus in all injected mice, with a pattern similar to that of Peak 1 Aβ derived from plaque-bearing APP tg mice (Fig. 8D and E, Supplementary Fig. 9). However, Peak 1 Aβ from AD1 and AD2 induced Aβ deposition in only 3 out of 5 mice injected and Peak 1 Aβ from AD3 did not induce Aβ deposition in any mouse. Furthermore, we examined the seeding capability of Peak 1 Aβ derived from Peak 1 Aβ-positive control brain NC2 and found that Peak 1 Aβ from NC2 induced Aβ deposition in 2 out of 4 mice injected (Fig. 8D and E, Supplementary Fig. 9). We quantified the area fraction of Aβ deposition in the Peak 1-Aβ-injected versus PBS-injected hippocampi and found that Peak 1 Aβ from AD4, AD5 and AD6 caused a significant increase in the area of Aβ deposition in the hippocampus, while Peak 1 Aβ from AD1, AD2 and NC2 showed a non-significant uptrend, and Peak 1 Aβ from AD3 did not cause any increase (Fig. 8F). Moreover, to investigate whether there was any correlation between the degree of seeding activity and neuropathological features, we analysed adjacent brain sections from the same autopsy cases by anti-Aβ immunohistochemistry (Supplementary Fig. 8B) and compared these findings with individual seeding efficiencies of Peak 1 Aβ. However, no clear correlation was detected between neuropathological features and seeding activity. These findings suggest that Peak 1 Aβ is present in the brains of patients with Alzheimer’s disease, not just in plaque-bearing APP tg mice; however, its ability to induce β-amyloidosis may vary among individuals.

## Discussion

In this study, we found that TBS-soluble >150 kDa Aβ oligomeric species (‘Peak 1 Aβ’) existing in the brains of plaque-bearing APP tg mice or patients with Alzheimer’s disease can induce Aβ deposition and spreading in the brain. Our findings indicate that Peak 1 Aβ represents a molecular culprit that contributes to the spatiotemporal spreading of Aβ deposition in the Alzheimer’s disease brain. Previous studies have reported the presence of small and large Aβ oligomers in the brains of patients with Alzheimer’s disease or APP tg mice.^5,16,28,29,33-36,43-45^ It has been also reported that inoculation of soluble lysate of Alzheimer’s disease brains leads to β-amyloidosis.^36,46,47^ However, what soluble Aβ oligomeric species contribute to the deposition and/or spreading of Aβ was incompletely understood. We demonstrate that injection of TBS-soluble Aβ species with a molecular weight of >150 kDa (Peak 1 Aβ) induced β-amyloidosis in the brain, whereas Peak 2 Aβ with a 50–70 kDa, and Peak 3 Aβ with a 10–20 kDa, failed to induce Aβ deposition in the brain (Fig. 1). In addition, formic-acid denaturation experiments showed that Aβ oligomers in the Peak 1 Aβ are essential for the induction of β-amyloidosis (Fig. 6). These data strongly suggest that Peak 1 Aβ is a critical Aβ species for the induction of β-amyloidosis. *In vitro* fibrillization assays have reported that Aβ protofibrils are a type of soluble Aβ oligomer with a molecular weight of 200–300 kDa^32,48^ and that Aβ protofibrils are conformers on-pathway towards Aβ fibrillization that serve as a template for Aβ fibrils.^32,49^ However, injection of micromolar concentrations of Aβ protofibrils into the brains of APP tg mice induced only minimal Aβ deposition.^13,20^ In contrast, we found that Peak 1 Aβ, which contains Aβx-42 at a concentration of ∼100 pM, induced substantial β-amyloidosis in the hippocampus. This finding suggests that Peak 1 Aβ has a considerably greater propensity to induce β-amyloidosis in the brain than Aβ protofibrils, despite having a similar molecular size. mAb158/lecanemab is a monoclonal antibody that targets Aβ protofibrils, and mAb158 is the murine version of lecanemab.^50^ mAb158 has also been shown to recognize TBS-soluble Aβ species with diameters of 25–40 and heights of 1.8–3.0 (estimated molecular size of 80–500 kDa) in plaque-bearing APP tg mice,^51,52^ suggesting that mAb158 may recognize Peak 1 Aβ. To evaluate potential similarities, particularly in higher-order structure, between protofibrils and Peak 1 Aβ, it is therefore important to examine the reactivity of mAb158 towards Peak 1 Aβ. In addition, passive immunization with mAb158 in APP tg mice has been shown to reduce brain Aβ protofibril levels and prevent Aβ plaque formation.^53^ Assessing whether administration of mAb158 in APP tg mice also reduces Peak 1 Aβ levels would further contribute to evaluating the similarity between Peak 1 Aβ and protofibrils. Recently, Stern *et al.*^54^ revealed that amyloid fibrils with an average length of ∼100 nm, positive for anti-Aβ protofibril antibody lecanemab, were present in ultracentrifugal supernatants obtained from Alzheimer’s disease brain extracts. However, in negative staining electron microscopy study, we did not observe any amyloid fibrils in the Peak 1 fraction (Supplementary Fig. 3). Further ultrastructural examination combined with immunolabeling using an anti-Aβ antibody is necessary to determine what is the primary Aβ structure in Peak 1 Aβ.

We observed that administration of Peak 1 Aβ into the hippocampus induced characteristic band-like Aβ deposits along the molecular layer 2 m.p.i. (Figure 4B) In contrast, administration of Peak 1 Aβ into the cisterna magna induced Aβ deposition within the walls of leptomeningeal arteries surrounding the brain 4 m.p.i. (Figure 2). These findings suggest the possibility that the patterns of various β-amyloidosis, such as senile plaques in the brain parenchyma and CAA around blood vessels, may be influenced by the routes through which Aβ seeds spread within the brain. The mechanism(s) by which Peak 1 Aβ exerts its seeding ability remains unclear. One possibility is that Peak 1 Aβ induces β-amyloidosis by promoting structural changes of Aβ in the brain. In the present *in vivo* seeding experiments, the injection of 2.5 μL of Peak 1 Aβ (about 100 pM of Aβ) triggered the formation of 150–200 nM of SDS-insoluble/formic-acid-soluble Aβ in the brain after 4 or 6 months (Fig. 4). Additionally, we found a positive correlation between Peak 1 Aβ levels and the insoluble Aβ levels in biochemical analyses of APP tg mouse brains (Fig. 3). These findings suggest that Peak 1 Aβ may have facilitated β-amyloidosis by interacting with Aβ in the brains of APP tg mice and promoting its structural conversion to an amyloid form. Another possibility is that Peak 1 Aβ could create a microenvironment that concentrates Aβ and promotes β-amyloidosis. In this study, the injection of Peak 1 Aβ in the hippocampus did not simply diffuse from the injection site to form Aβ plaques but rather generated distinctive band-like Aβ deposits along the molecular layer of the hippocampal dentate gyrus. This area is known to exhibit Aβ deposits presumably resulting from Aβ released by the axon terminals of perforant pathway neurons that project from the entorhinal cortex.^24,55^ This observation suggests that Peak 1 Aβ provides a microenvironment with extracellular matrix molecules that fosters β-amyloidosis in the brain.

Importantly, we found that Peak 1 Aβ is present in the brains of patients with Alzheimer’s disease, with a similar ability to induce β-amyloidosis in the brains of young APP tg mice; however, this seeding capacity appears to be heterogenous across individuals (Fig. 8). This is reminiscent of the heterogeneity of tau seeding capacity across Alzheimer’s disease donors^56^ and consistent with a recent study reporting that intracerebral injection of brain homogenates from patients with Alzheimer’s disease.^57^ Thus, these findings suggest that the seeding ability of Peak 1 Aβ in the brains of patients with Alzheimer’s disease to induce β-amyloidosis may differ from case to case. Recently it has been reported that the Aβ43 aggregates exhibited higher seeding capability than Aβ42, Aβ40 and Aβ38.^58^ Further investigation of Aβx-43 in Peak 1 fractions is required. Prior *in vivo* seeding experiments have also reported that the seeding activity in the brain extracts from APP tg mice peaks at the early stage of β-amyloidosis.^59^ In addition, there is evidence for a longevity-dependent decrease in prion-like activity in brain extracts from patients with Alzheimer’s disease in Aβ-cellular assays.^60^ In this study, we found Peak 1 Aβ derived from Peak 1 Aβ-positive control brain (NC2) also induced β-amyloidosis, however, there was no significant difference in the Aβ-positive areas compared to the PBS-injection (Fig. 8D and E), suggesting that the levels of Peak 1 Aβ might have begun to increase in the brain before the onset of the diseases. These results also suggest that the seeding activity of brain extracts may differ depending on the Aβ deposition phase. To shed more light on the factors that determine the differences in the seeding ability of Peak 1 Aβ in Alzheimer’s disease patient brains, peptide mapping of Aβ in Peak 1 Aβ fractions by mass spectrometry (MS), identification of Peak 1 Aβ protein interactors by liquid chromatography-MS/MS, would be required.

It remains unclear which Aβ species are induced to deposit by injection of Peak 1 Aβ. The APP tg A7 line produces higher levels of Aβ42 than Aβ40,^11^ and consistent with this, the amount of insoluble Aβ42 was greater than that of Aβ40 in the biochemical analysis (Fig. 4). In contrast, immunohistochemical analyses using C-terminal-specific antibodies revealed only weak Aβ42 immunoreactivity, whereas Aβ40 and Aβ43 were scarcely detected (Supplementary Fig. 4). This discrepancy may be attributable to epitope masking at the C-terminus of Aβ and therefore further protein analyses of insoluble Aβ, including MS-based approaches, will be required in future studies.

Although soluble Aβ oligomers in the brains of patients with Alzheimer’s disease or APP tg mice are thought to cause neurotoxicity or synaptic dysfunction,^27,33,34,61-63^ whether this is also the case for the Aβ oligomers contained in Peak 1 Aβ remains an open question. In our *in vivo* seeding experiments, we observed astrocytosis or microgliosis around the Aβ deposits induced by Peak 1 Aβ and our RNA-seq analysis revealed 32 genes differentially expressed after induction of β-amyloidosis including upregulated glial genes and downregulated neuronal genes (Fig. 7), suggesting that Peak 1 Aβ may elicit local toxicity through the induction of β-amyloidosis. Among the downregulated neuronal genes, *Nptx2* encodes neuronal pentraxin 2, a protein involved in synaptic function, and it has been reported that the level of NPTX2 was reduced in the cerebrospinal fluid of patients with Alzheimer’s disease, autosomal dominant Alzheimer’s disease, or adults with Down syndrome,^64-67^ suggesting that Aβ deposition induced by Peak 1 Aβ may induce the expression changes of synaptic genes in the brain.

In summary, we have identified the Aβ species, collectively termed ‘Peak 1 Aβ’, which drive the seeding, deposition and spreading of Aβ in the brain and recapitulate the early stages of Aβ plaque formation, including reactive astrogliosis and microgliosis. These findings will give us clues to understand the detailed process of formation of Aβ pathology in the Alzheimer’s disease brain, and further provide a novel therapeutic target for Alzheimer’s disease by suppressing Aβ deposition and spreading through immunotherapy for Peak 1 Aβ.

## Supplementary Material

fcag188_Supplementary_Data

## Data Availability

RNA-seq data are deposited in DDBJ (https://ddbj.nig.ac.jp/search/entry/bioproject/PRJDB39935). The datasets and materials used during the current study are available from the corresponding authors upon reasonable request.
