# Supplementary material for: Soluble high-molecular-weight amyloid-β species derived from amyloid-β-laden brains induce cerebral β-amyloidosis
Source: Brain Commun. 2026 Jul 20;8(4):fcag188. doi: 10.1093/braincomms/fcag188 (PMC13381059; doi:10.1093/braincomms/fcag188)
Supplement: fcag188_Supplementary_Data [file fcag188_supplementary_data.docx]

Supplementary material

Soluble high-molecular-weight amyloid-β species derived from amyloid-β-laden brains induce cerebral β-amyloidosis

Mayu Kashiwagi-Hakozaki, Hirokazu Uchigami, Yasushi Naka, Asuka Kokawa, Tatsuo Mano, Junki Cho, Kaoru Yamada, Akinori Miyashita, Norikazu Hara, Takeshi Ikeuchi, Alberto Serrano-Pozo, Matthew P Frosch, Bradley T Hyman, Tatsushi Toda, Masashi Fukayama, Tetsuo Ushiku, Tomoko Wakabayashi, Takeshi Iwatsubo, and Tadafumi Hashimoto

**Supplementary Experimental Procedures**

***Negative staining electron microscopy***

3 μL of the peak 1 fraction or TBS-insoluble fraction were spread on 250-mesh elastic-carbon-coated grid (ELS-C10, STEM Cu100P grid, Okenshoji). Following a hydrophilization using a grow discharger (6 mA, 30 sec), samples were negatively stained with 2% (w/v) phosphotungstic acid (pH=7.0), and viewed in an electron microscope (JEOL JEM-1400Flash).

***RNA-sequencing (RNA-seq)***

Total RNA was isolated from mouse hemibrain tissue using TRIzol reagent (Thermo Fisher Scientific), and 500 ng of total RNA was used to prepare RNA-Seq libraries using the TruSeq Standed mRNA Library Prep Kit (Illumina). The libraries were then sequenced on the NextSeq500 next-generation sequencer (Illumina), and each library yield an average of 42 million 75 bp paired-end reads. The sequenced reads were mapped to the mouse reference genome (GRCm38: Ensembl release 102) using *Salmon* v1.4.0. Differential gene expression analysis was performed using *DESeq2* v1.30.1. Lowly expressed genes (mean normalized read counts < 10) were filtered out. P-Values were calculated using the Wald test and adjusted for multiple testing using the Benjamini-Hochberg procedure. Genes with adjusted P-values less than 0.05 (5% false discovery rate) were considered significant.

Brain single-nucleus RNA-seq (snRNA-Seq) of the prefrontal cortex from AD and controls was obtained from publicly available databases (<https://www.synapse.org/#!Synapse:syn18485175>). ^1^ The use of this dataset for the project has been approved by Synapse on 07/10/2022. The filtered dataset with pre-calculated tsne coordinates were used in this study. This dataset includes 17,926 genes profiled in 75,060 nuclei, and eight major cell types (excitatory neurons, inhibitory neurons, astrocytes, oligodendrocytes, microglia, oligodendrocyte progenitor cells, pericytes and endothelial cells), were annotated. The dataset was analyzed using the Seurat R package (V4.0).^2^ R was used for the analysis. The raw reads counts were long-normalized and multiplied a scale factor 10,000 and scaled so that the mean expression and the variance across cells were 0 and 1, respectively. Signature scores for the genes of interest were computed using the AUCell method (v.1.18.1).^3^

**References**

1. Mathys H, Davila-Velderrain J, Peng Z, *et al*. Single-cell transcriptomic analysis of Alzheimer’s disease. *Nature*. 2019;570:332-337.
2. Hao Y, Hao S, Andersen-Nissen E, *et al*. Integrated analysis of multimodal single-cell data. *Cell*. 2021;184:3573-3587.
3. Aibar S, González-Blas CB, Moerman T, *et al*. SCENIC: single-cell regulatory network inference and clustering. *Nat Method*. 2017;14:1083-1086.

**Supplementary Figure 1**

**
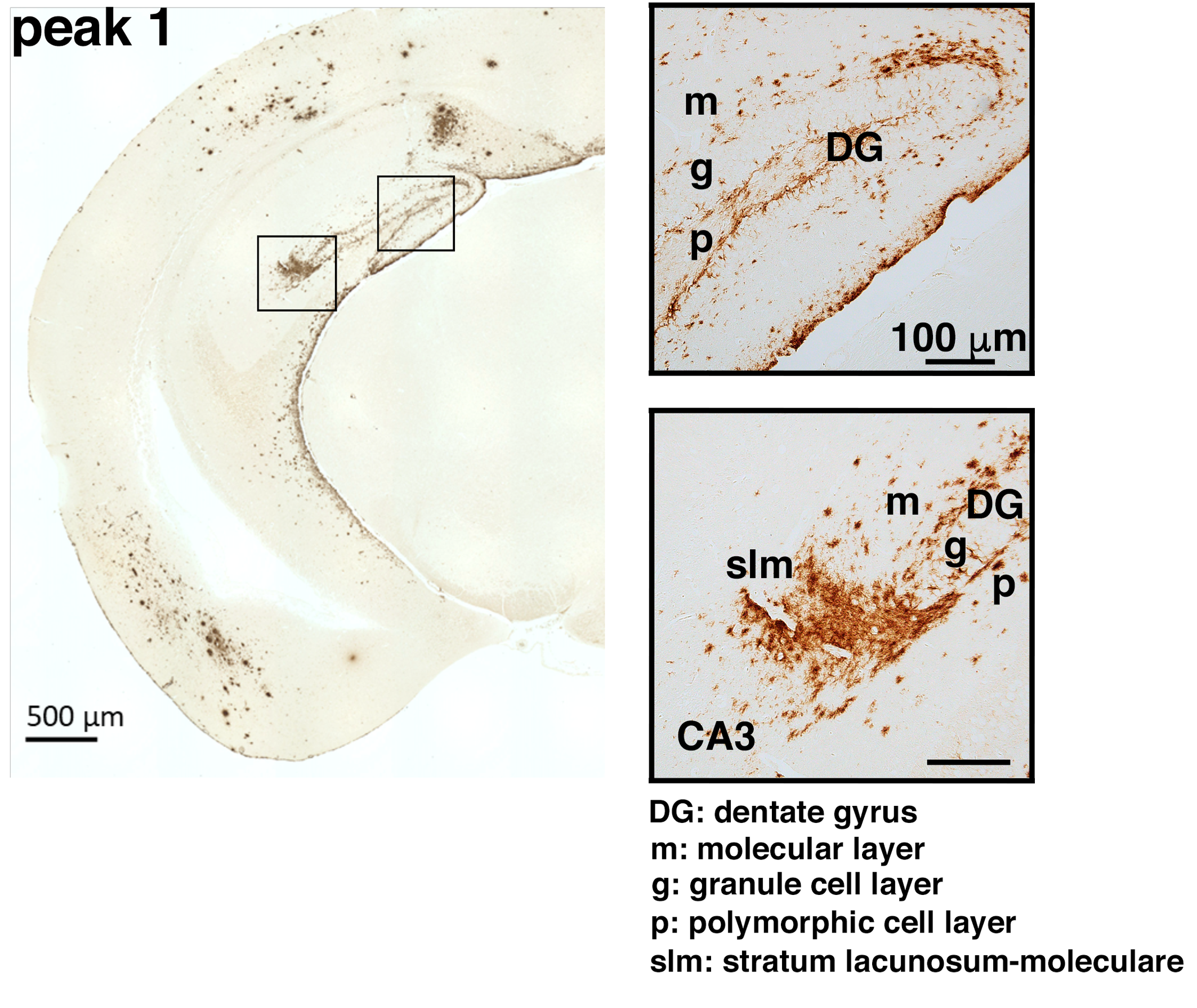
**

**Supplementary Figure 1 Enlarged views of β-amyloidosis induced by peak 1 amyloid-β (Aβ).**

Immunohistochemistry of Aβ precursor protein transgenic (APP tg) mice injected with peak 1 Aβ from the brains of 18-19-month-old APP tg mice using the anti-Aβ antibody 82E1 (left panel). Scale bar shows 500 μm. High magnification images (insets in left panel) are shown in right panels. Scale bar shows 100 μm. m: molecular layer, g: granule cell layer, p: polymorphic cell layer, slm: stratum lacunosum-moleculare.

**Supplementary Figure 2**

**
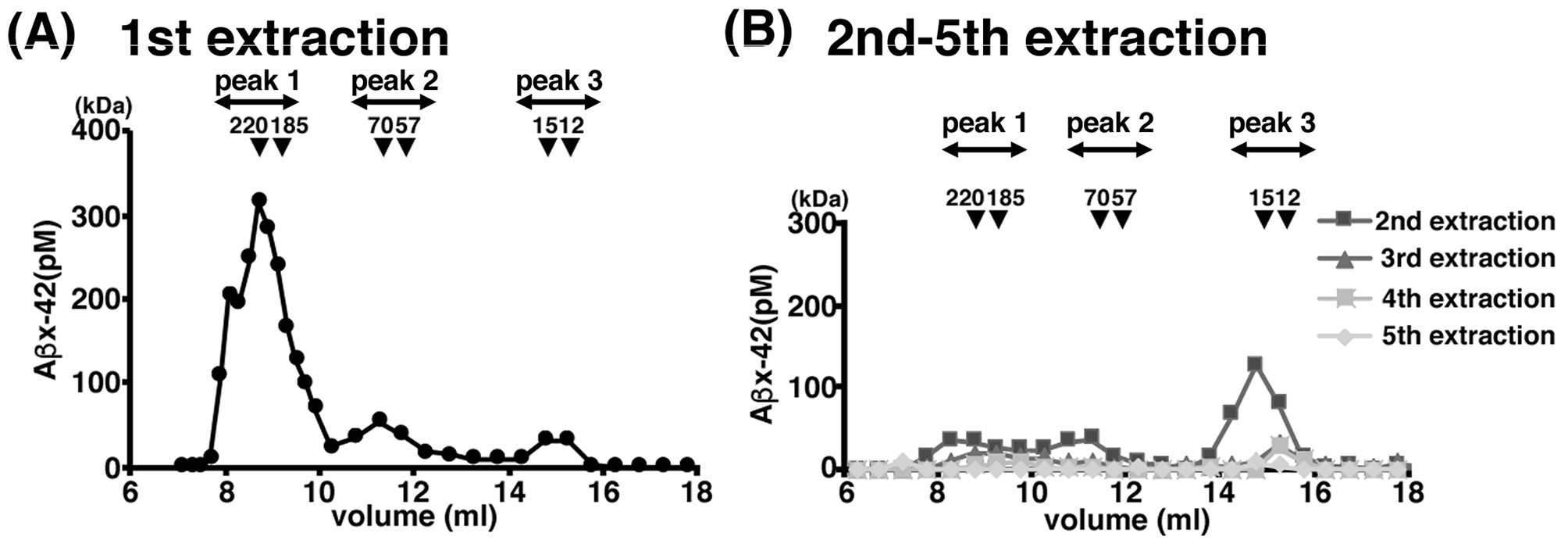
**

**Supplementary Figure 2 Separation of repeated extraction samples of Tris-buffered saline (TBS)-soluble fraction of amyloid-β (Aβ) precursor protein transgenic (APP tg) mice by size-exclusion chromatography (SEC).**

(**A**, **B**) Separation of repeated extraction samples of TBS-soluble fraction from 18-19-month-old APP tg mice by SEC (1st extraction in **A**, 2^nd^-5^th^ extraction in **B**). The concentration of Aβx-42 in the first extraction sample is shown in the left panel, and the concentration of Aβx-42 in the second, third, fourth, or fifth extraction sample is shown in the right panel. Estimated molecular weight (kDa) is indicated at the top (arrowheads). Each datapoint represents Aβ levels measured in repeated TBS extracts from a single brain. Data are derived from a single experiment and are presented as representative results (*n*=1 biological replicate).

**Supplementary Figure 3**

**
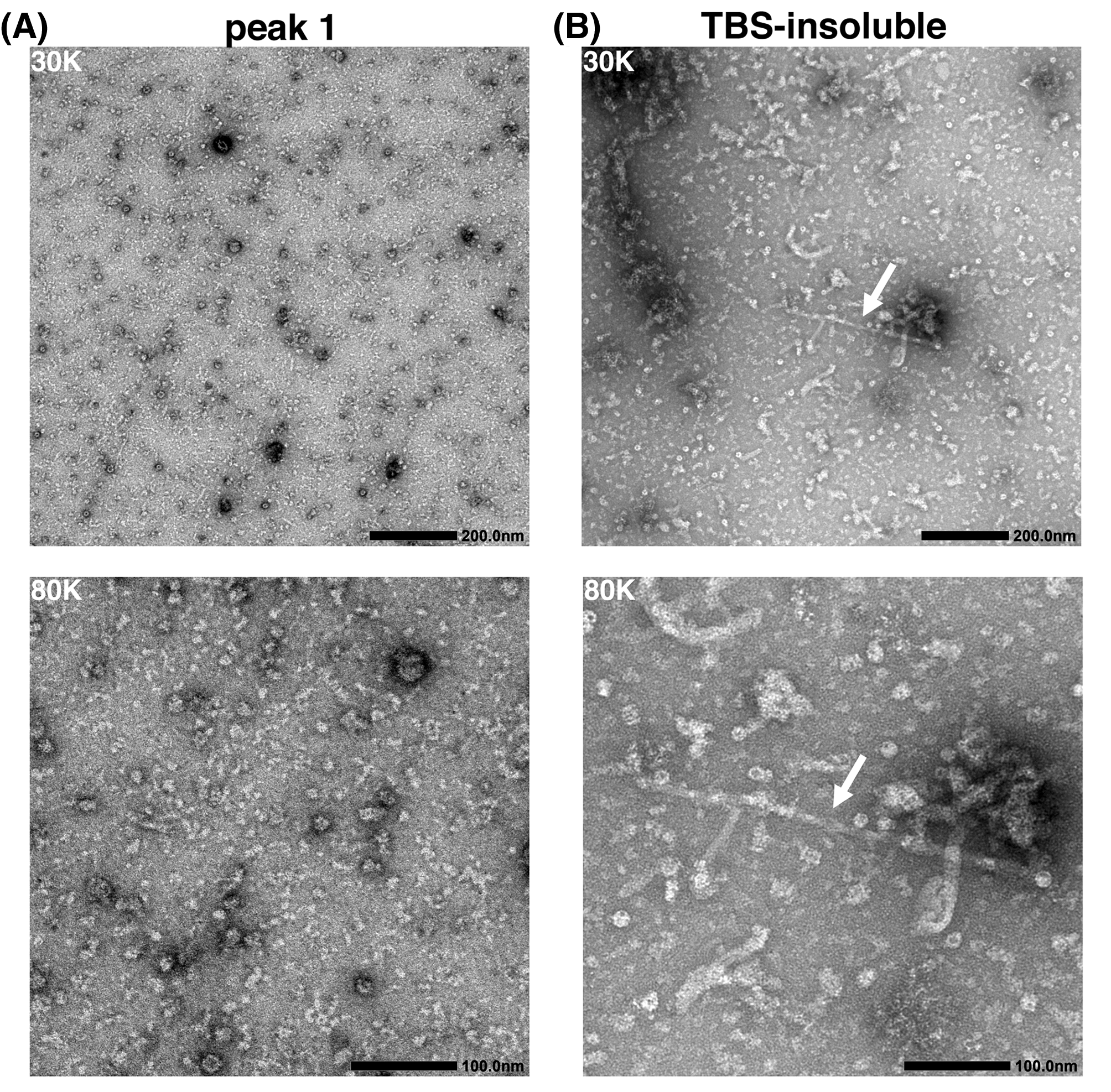
**

**Supplementary Figure 3 Negative staining electron microscopy analyses of peak 1 and Tris-buffered saline (TBS)-insoluble fractions from amyloid-β (Aβ) precursor protein transgenic (APP tg) mice.**

(**A**, **B**) Representative low magnification (x30,000, scale bar shows 200 nm, upper panels) and high magnification (x80,000, scale bar shows 100 nm, lower panels) images of negative staining electron microscopy of peak 1 fractions (**A**) or TBS-insoluble fraction (**B**) from the brains of 24-month-old APP tg mice. Arrows indicate amyloid fibrils.

**Supplementary Figure 4**

**
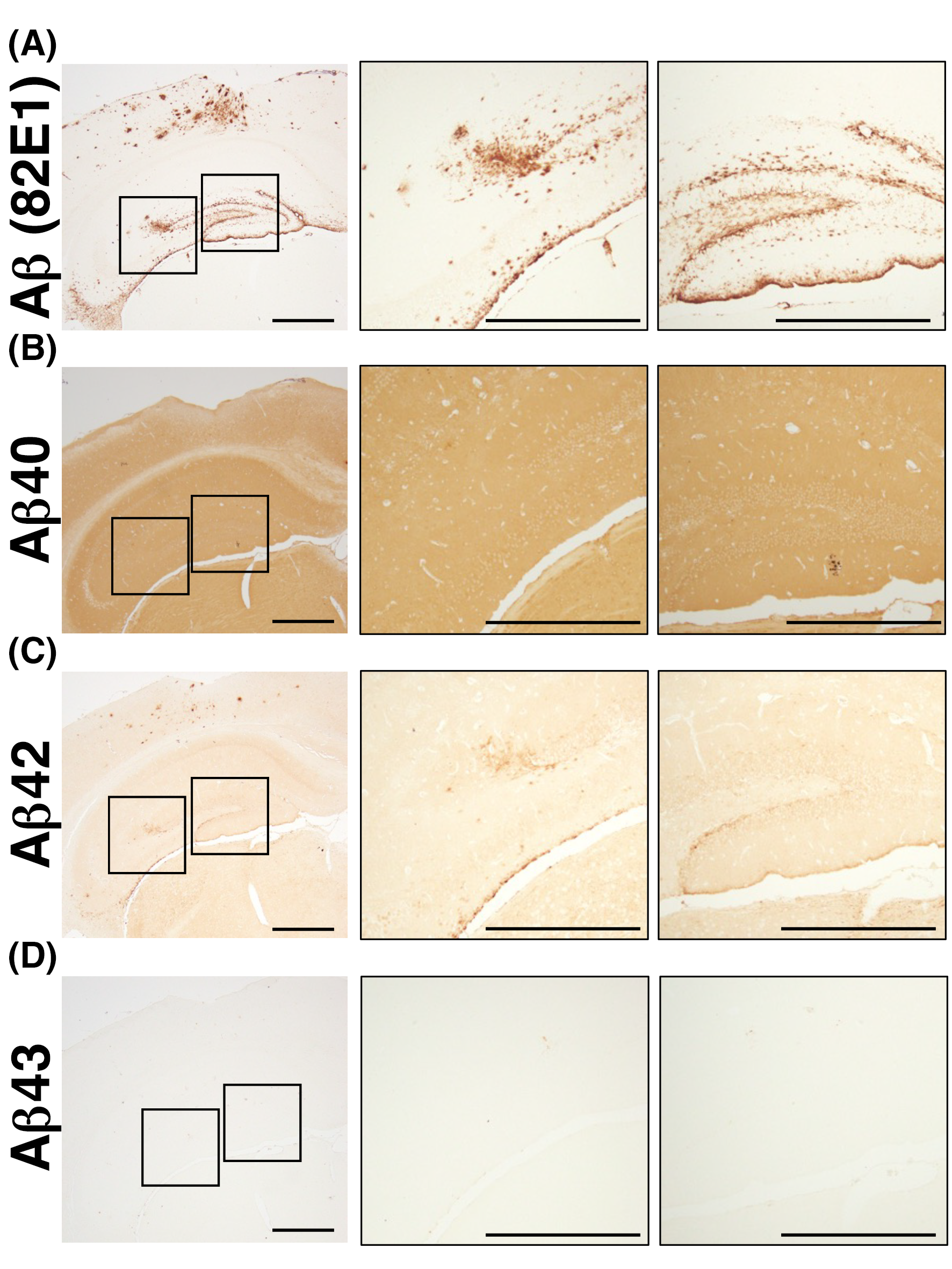
**

**Supplementary Figure 4 Immunohistochemical analysis of brains of amyloid-β (Aβ) precursor protein transgenic (APP tg) mice injected with peak 1 Aβ using carboxy-terminus specific antibodies.**

(**A**-**D**) Immunohistochemical analysis of the brains of APP tg mice injected with peak 1 Aβ derived from the brain of 29-month-old APP tg mouse using an anti-human Aβ antibody 82E1 (**A**), an anti-Aβ40 specific antibody BA27 (**B**), an anti-Aβ42 specific antibody BC05 (C), or an anti-Aβ43 specific antibody C43 (**D**). The scale bar indicates 500 μm.

**Supplementary Figure 5**

**
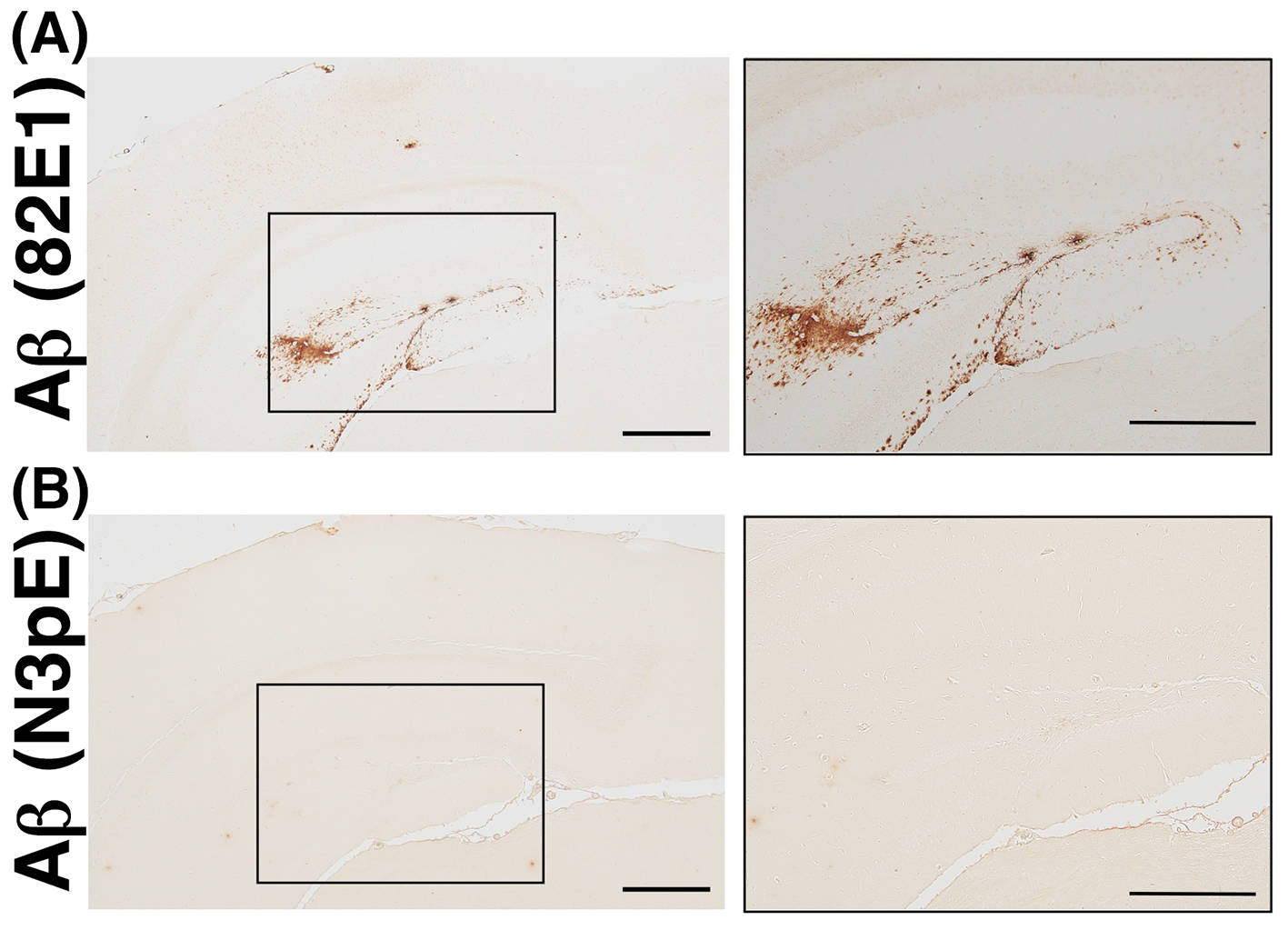
**

**Supplementary Figure 5** **Immunohistochemical analysis of brains of amyloid-β (Aβ) precursor protein transgenic (APP tg) mice injected with peak 1 Aβ using pyroglutamate-modified Aβ at position 3.**

(**A**, **B**) Immunohistochemical analysis of the brains of APP tg mice injected with peak 1 Aβ derived from the brain of 25-month-old APP tg mouse using an anti-human Aβ monoclonal antibody 82E1 (**A**), or a polyclonal antibody against pyroglutamate-modified Aβ at position 3 (N3pE) (**B**). The scale bar indicates 500 μm.

**Supplementary Figure 6**

**
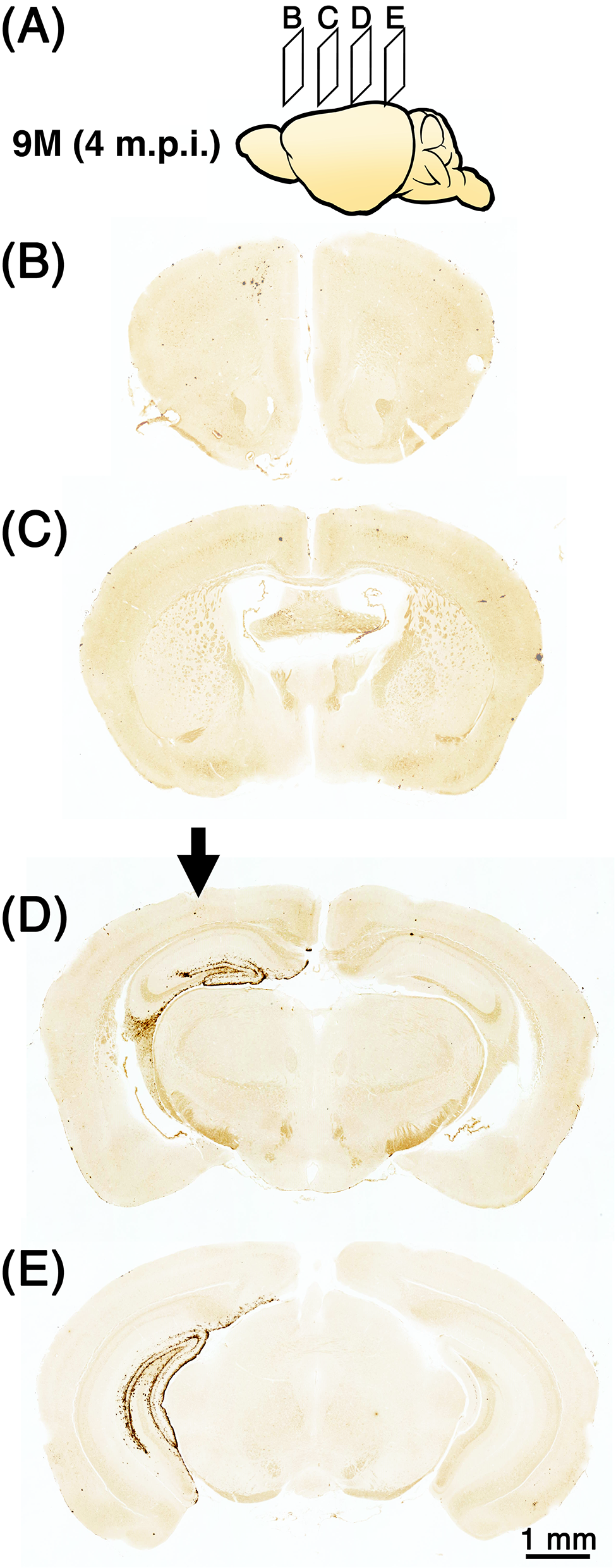
**

**Supplementary Figure 6 Anterior-posterior images of β-amyloidosis induced by peak 1 amyloid-β (Aβ).**

(**A**) Schematic of anterior-posterior coordinates of the mouse brains. (**B-E**) Immunohistochemistry of the anterior-to-posterior sections of the brains of 29-month-old Aβ precursor protein transgenic mice injected with peak 1 Aβ (left hippocampus) or phosphate-buffered saline (right hippocampus) using anti-Aβ antibody 82E1. Arrow in (**D**) indicates the injection site. Scale bar shows 1 mm.

**Supplementary Figure 7**

**
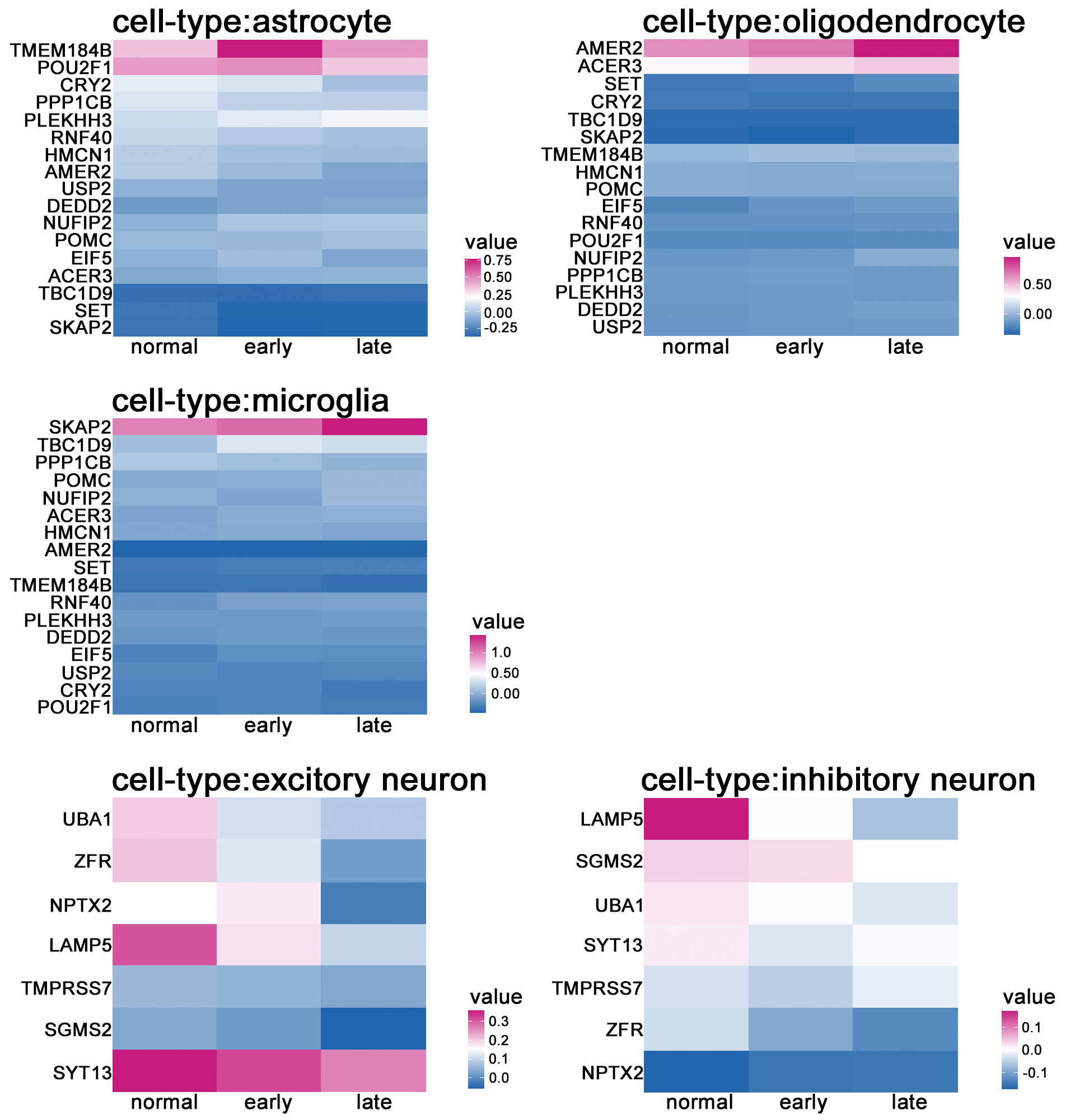
**

**Supplementary Figure 7 Cell type-specific and disease progression-dependent gene expression analysis of up- or down-regulated genes.**

Gene expression analysis of up- or down-regulated genes per astrocyte, oligodendrocyte, microglia, excitatory neuron, or inhibitory neuron subclass in the dorsolateral prefrontal cortex of normal (Braak stages 0, 1 and 2), early (Braak stages 3 and 4) or late (Braak stages 5 and 6) Alzheimer’s disease stages using the public single nucleus RNA-sequeincing dataset from Mathys *et al.*^1^

**Supplementary Figure 8**

**
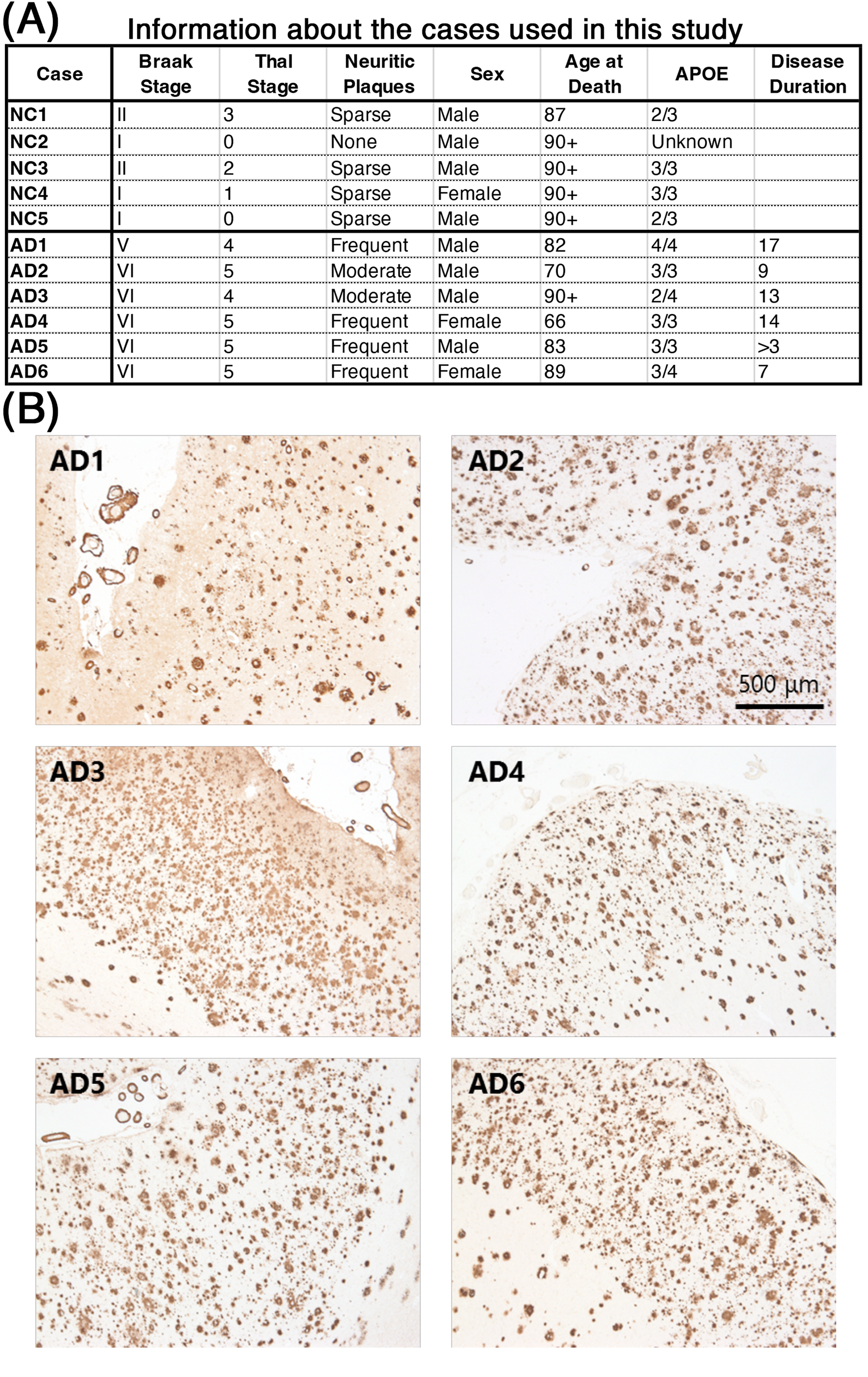
**

**Supplementary Figure 8 Information on the cases used in this study.**

(**A**) Information on the Alzheimer’s disease (AD) and control cases used in this study. (**B**) Representative images of immunohistochemical staining of AD cases using anti-amyloid β antibody. Scale bar shows 500 μm.

**Supplementary Figure 9**

**
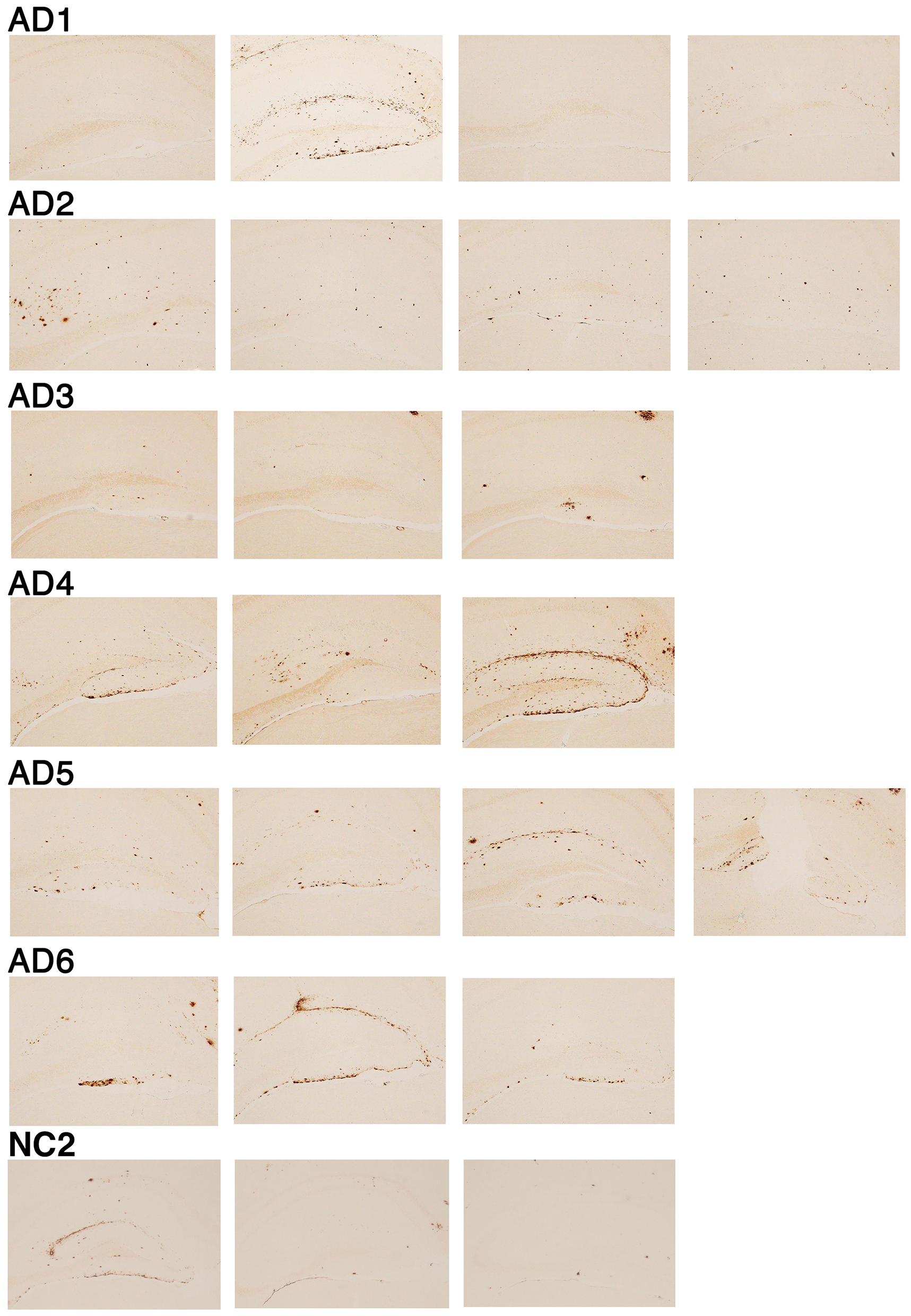
**

**Supplementary Figure 9 Immunohistochemical analyses of the brains of amyloid-β (Aβ) precursor protein transgenic (APP tg) mice injected with peak 1 Aβ from Alzheimer’s disease (AD) brains and a control brain.**

Immunohistochemical analyses of the brains of APP tg mice injected with peak 1 Aβ from brains of AD1, AD2, AD3, AD4, AD5, AD6, and NC1. Representative images of Aβ staining corresponding to the quantification shown in Figure 8F. These images illustrate that the extent of Aβ deposition varies depending on the source of the AD patient brain used for induction.
